# Mertk promotes early microglial-mediated synaptic engulfment in Alzheimer's disease

**DOI:** 10.7150/thno.116797

**Published:** 2026-01-01

**Authors:** Yifan Wu, Yuanteng Fan, Shisan Bao, Yinghao Song, Ruizhu Wang, Jiaxuan Wu, Xuan Liu, Jingwen Jin, Lingchen Kong, Baohua Hou, Peiyu Liang, Taoxiang Chen, Wanhong Liu, Biwen Peng, Fanggang He, Ying Zhou, Jian Xu, Yun Chen, Song Han, Jun Yin, Xiaohua He

**Affiliations:** 1 School of Basic Medical Sciences, Taikang Medical School, Wuhan University, 185 Donghu Road, Wuhan, 430071, China.; 2 Hubei Province Key Laboratory of Developmentally Originated Disease, School of Basic Medical Sciences, Taikang Medical School, Wuhan University, 185 Donghu Road, Wuhan, 430071, China.; 3 Department of Neurology, Zhongnan Hospital of Wuhan University,169 Donghu Road, Wuhan, 430071, China.; 4 Department of Pathology, The University of Sydney, NSW, 2006, Australia.; 5 College of Medicine, Henan Polytechnic University, 142 Jiefang Middle Road, Jiaozuo, 454000, China.; 6 Wuhan University (School of Basic Medical Sciences)-Weifang Joint Research Center for Pediatric Neurological Diseases and Innovation and Transformation, 185 Donghu Road, Wuhan, 430071, China.; 7 Hubei Province Key Laboratory of Allergy and Immune Related Disease, School of Basic Medical Sciences, Taikang Medical School, Wuhan University, 185 Donghu Road, Wuhan, 430071, China.; 8 WeiFang Maternal and Child Health Hospital, 12007 Yingqian Road, Weifang, 261000, China.; 9 State Key Laboratory of Oral & Maxillofacial Reconstruction and Regeneration, School & Hospital of Stomatology, Wuhan University, 237 Luoyu Road, Wuhan, 430079, China.

**Keywords:** Mertk, microglia, synaptic engulfment, Alzheimer's disease, PPARγ

## Abstract

**Rationale**: Synaptic deficits occur prior to the emergence of Aβ plaques and tau pathology in Alzheimer's disease (AD). Dysregulated microglia excessively prune synapses, leading to synaptic loss. While microglia phagocytic receptor Mertk participates in synaptic pruning, the role of Mertk in driving early synaptic loss in AD remains elusive.

**Methods:** Single-cell RNA sequencing (scRNA-seq) was used to analyze transcriptional changes of microglia in early stage of AD mice. Mertk-mediated synaptic engulfment was investigated both *in vivo* and *in vitro*.

**Results:** Phagocytic-associated microglia with upregulated Mertk were identified in the early stage of AD mice. Dysregulated synaptic pruning by microglia caused hippocampal synaptic loss and memory deficits in two AD mouse models. Notably, Mertk knockout or antagonist treatment reversed excessive synapse elimination by microglia. Mechanistically, Aβo-induced PPARγ promoted Mertk transcription, mediating microglial phagocytosis of synapses.

**Conclusions:** Collectively, our findings suggest that PPARγ-regulated, Mertk-mediated microglial synaptic engulfment contributes to early synaptic loss in AD, highlighting microglial Mertk as a potential therapeutic target for AD.

## Introduction

Alzheimer's disease (AD) is the most prevalent neurodegenerative disorder in elderly individuals, characterized by the accumulation of amyloid-beta (Aβ) and tau pathology, which contribute to cognitive decline and memory impairment 1, 2. The pathogenesis of AD remains incompletely understood, and the number of individuals affected continues to rise each year 3, 4. The optimal therapeutic window for intervention is during the early stage of the disease, particularly in individuals with mild cognitive impairment (MCI) 5-7. Currently, an increasing number of novel therapeutics target the early pathological features of AD, specifically Aβ immunotherapy 5, 8. Drugs like *aducanumab* 9 and *lecanemab* 10 have shown certain therapeutic effects in early stage AD patients, but offer limited cognitive improvement and are associated with high costs and side effects, including ARIA-edema (ARIA-E) and ARIA-hemorrhage (ARIA-H) 11-13. The precise mechanisms underlying AD development, particularly the involvement of key molecules, remain unclear. Thus, there is an urgent need for early diagnosis and the identification of effective therapeutic targets.

Synapse loss is a significant pathological feature of AD that correlates with cognitive decline, often manifesting in the early stage of the disease—prior to the formation of amyloid plaques, neurofibrillary tangles 14-18. Consequently, synaptic preservation represents a critical early therapeutic strategy. Microglia, the resident immune cells of the brain, play essential roles in various physiological and pathological processes, acting as the "clean-up masters" of the central nervous system (CNS). Microglia are crucial for developmental synaptic remodeling, as they interact with and prune underdeveloped synapses in healthy CNS 19-22. Furthermore, microglia can become excessively activated in certain neurological disorders, leading not only to the pruning of underdeveloped synapses but also to excessive phagocytosis of healthy synapses 23-27. Such abnormal microglial activity disrupts normal neural circuitry, ultimately resulting in cognitive impairments.

Previous studies have identified several signaling molecules involved in microglial synaptic pruning under pathological conditions, including components of the complement pathway 28, 29, CX3CR1 22, 30, SIRPα 31, 32 and TREM2 33, 34. Synaptic pruning mediated by these molecules is primarily observed at the later stages of AD, making them unsuitable as targets for early diagnosis and treatment. Some pruning mechanisms, such as the complement cascade, involve complex signaling pathways and contribute to various pathological processes in AD. This complexity complicates the design of precise therapeutic interventions. Moreover, the molecular mechanisms involved in synapse elimination by microglia in the early stage of AD remain unknown. Therefore, there is an urgent need to explore new potential targets for triggering phagocytosis.

Mertk (Mer tyrosine kinase) is a member of the TAM (TYRO3/AXL/MERTK) receptor tyrosine kinase family 35, which can be activated by two ligands, growth arrest-specific gene 6 (Gas6) and Protein S (Pros1) 36-39. As a phagocytic receptor, Mertk is predominantly expressed in microglia within the CNS, where it mediates the clearance of apoptotic cells 40, myelin debris 41 and synapses 42. Mertk plays a critical role in synaptic pruning by recognizing 'eat-me' signals, such as phosphatidylserine on apoptotic fragments, during various physiological and pathological processes, including stroke 23, stress 43, seizures 44 and retinitis pigmentosa 45. Additionally, Mertk has been implicated in modulating microglial recognition and integration of amyloid plaques. The formation of dense-core plaques limits the dissemination of toxic pre-plaque Aβ oligomers (Aβo) throughout the brain, reducing cognitive deficits of fear acquisition 46. However, the role of Mertk in the early stage of AD — the presence of Aβo before plaque formation 28, 47 — remains insufficiently elucidated, particularly with regard to its function as a phagocytic receptor in synaptic modulation.

In the current study, we primarily focus on the early stage of AD, characterised by abnormalities in Aβ oligomers (Aβo), while plaques have not yet formed. Our study demonstrates that Mertk mediates excessive synaptic phagocytosis by microglia, leading to early synaptic loss and memory deficits in AD, providing novel insights into the underlying role of Mertk in the early stage of AD. Moreover, Mertk^-/-^ mice exhibited significantly reduced microglial phagocytosis of synapses in response to Aβo stimulation, along with improved behavioral outcomes. Further research indicated that the heightened reactivity of Mertk under Aβo stress is regulated by the transcription factor PPARγ. Taken together, our results suggest that microglial Mertk is involved in synaptic loss before plaque formation in early stage of AD, offering a new strategy for the prevention and treatment of AD.

## Results

### Increased phagocytic-related microglia in early-stage AD mice

**Figure 1:**
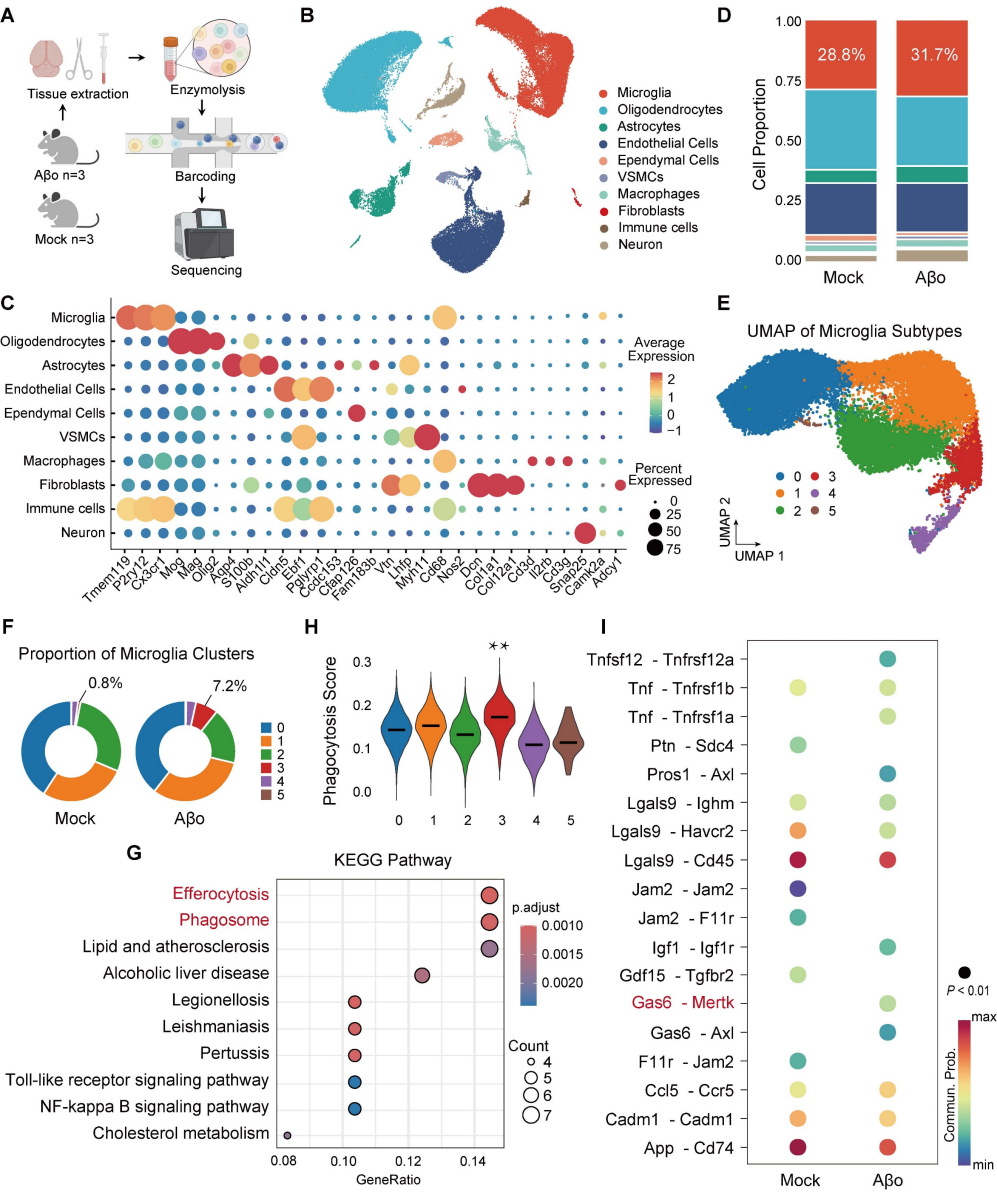
** Microglia exhibit a phagocytic-like phenotype accompanied by upregulation of the Gas6-Mertk signaling pathway in Aβo-treated mice.** (**A**) Schematic of the experimental workflow for single-cell RNA sequencing (scRNA-seq) of whole-brain tissue from Aβo-treated mice and mock-treated controls (*n* = 3 mice/group). (**B**) Uniform manifold approximation and projection (UMAP) representation of all cells (n = 67,876) from scRNA-seq, colored by annotated cell type. Data are shown after quality control and batch correction. (**C**) UMAP visualization of the entire scRNA-seq dataset, colored by marker gene expression levels for each cell type. (**D**) Stacked bar chart displaying the relative proportions of different cell populations in Aβo-treated mice and mock-treated controls. (**E**) UMAP visualization of microglia, colored by clusters and annotated accordingly. (**F**) Pie charts showing the percentage distribution of each microglial cluster in Aβo-treated and mock-treated mice. (**G**) KEGG pathway analysis of upregulated differentially expressed genes in microglia from Aβo-treated and mock-treated mice. (**H**) Characteristic gene analysis revealed the correlation between the top-ranked genes and phagocytosis in six clusters of microglia. **P < 0.01. (**I**) Cell-cell communication analysis of differentially expressed receptor-ligand interactions in cluster 3 microglia from Aβo-treated and mock-treated mice.

We used an AD model with intracerebroventricular (ICV) injection of Aβ1-42 oligomers to simulate the early stage of AD, where Aβ is present but plaques have not yet formed. To investigate the transcriptional state of microglia during Aβo challenge in the early stage of AD, we performed single-cell RNA sequencing (scRNA-seq) on whole-brain tissues from Aβo-treated mice and mock-treated controls, profiling 67,876 cells and identifying 10 distinct cell clusters (Figure 1A-B). Gene expression analysis categorized these cells into groups such as microglia, oligodendrocytes, and astrocytes, with cell identities confirmed by specific marker genes (Figure 1B-C). Among these cell types, the proportion of microglia increased in Aβo-treated mice (31.7%) compared to that in mock-treated controls (28.8%), indicating elevated microglial activity in response to Aβo (Figure 1D). We further clustered the microglia into six distinct groups (clusters 0-5), with cluster 3 showing a notable increase in proportion in Aβo-treated mice (7.2%) compared to mock-treated controls (0.8%) (Figure 1E-F). KEGG pathway analysis of upregulated differentially expressed genes revealed that microglia in Aβo-treated mice exhibited upregulation of pathways related to phagocytosis and efferocytosis, compared to mock-treated controls (Figure 1G). Furthermore, characteristic gene analysis showed that the top-ranked genes in cluster 3 microglia were more closely associated with phagocytosis than those in other clusters (Figure 1H). KEGG and GO enrichment analyses revealed the activation of endocytosis and phagocytosis-related pathways in cluster 3 microglia (Figure S1A-B). To further investigate the key molecular players in the enhanced phagocytic activity of microglia in the early stage of AD, we identified receptor-ligand interactions in cluster 3 microglia. The results indicated significant enrichment of the Gas6-Mertk signaling pathway in cluster 3 microglia of Aβo-treated mice relative to mock-treated controls (Figure 1I).

### Upregulation of Mertk in phagocytosis-related microglia during the early stage of AD

**Figure 2:**
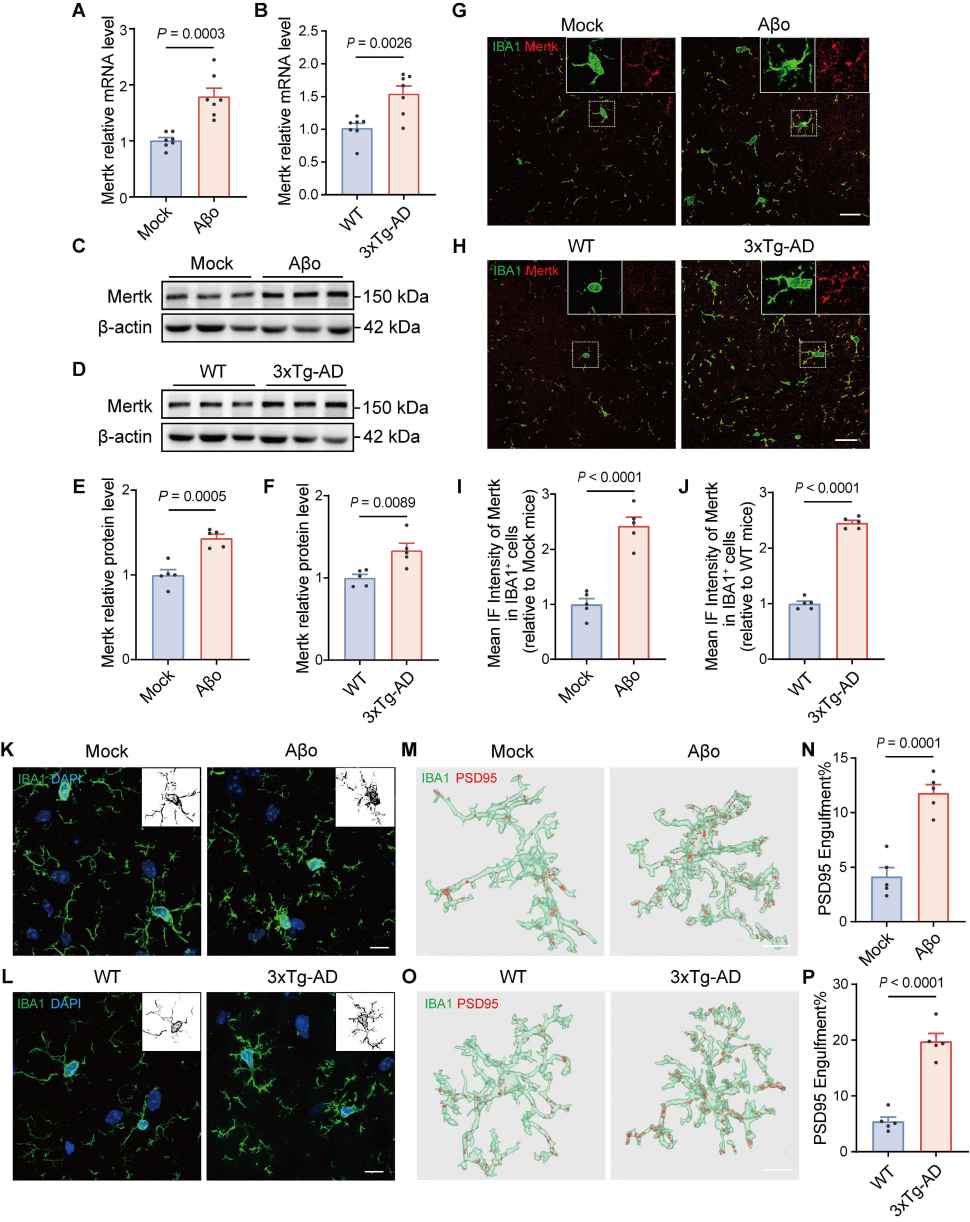
** Mertk is upregulated in phagocytic microglia of both Aβo-induced AD mice and 3xTg-AD mice.** (**A, B**) Quantification of Mertk mRNA levels by qPCR in (A) Aβo-treated and mock-treated mice, and (B) 4-month-old 3xTg-AD transgenic mice and age-matched WT controls. *n* = 7 mice/group; two-tailed unpaired *t*-test. (**C-F**) Western blot analysis of Mertk protein levels in (C, E) Aβo-treated and mock-treated mice, and (D, F) 4-month-old 3xTg-AD transgenic mice and WT controls. *n* = 5 mice/group; two-tailed unpaired *t*-test. (**G-J**) Immunostaining and quantification of Mertk co-labeled with the microglial marker IBA1 in the hippocampus of (G, I) Aβo-treated and mock-treated mice, and (H, J) 4-month-old 3xTg-AD and WT mice. Scale bar, 20 μm. *n* = 5 mice/group; 6-8 fields per mouse were analysed using two-tailed unpaired *t*-test. (**K, L**) Microglial morphology visualized by immunostaining for IBA1 (green) and DAPI (blue) in the hippocampus of (K) Aβo-treated and mock-treated mice, and (L) 4-month-old 3xTg-AD and WT mice. Scale bar, 10 μm. Representative grayscale images highlight microglial processes. (**M-P**) Three-dimensional reconstructions and surface renderings of PSD95^+^ puncta within IBA1-positive microglia in the hippocampus of (M, N) Aβo-treated and mock-treated mice, and (O, P) 4-month-old 3xTg-AD and WT mice. Scale bar, 10 μm. *n* = 5 mice/group; 6-8 microglia per mouse were analysed using one-way ANOVA with Dunnett's multiple comparisons test. Data are presented as means ± SEM.

To validate enhanced phagocytic microglia and an upregulated Mertk pathway in the early stage of AD, we investigated two AD mouse models: Aβo-induced AD mice and 4-month-old 3xTg-AD transgenic mice. Furthermore, we confirmed that 4-month-old 3xTg-AD mice do not exhibit amyloid plaques at this age, as demonstrated by 6E10 staining, in contrast to 8-month-old 3xTg-AD mice (Figure S2A). Mertk was significantly upregulated at both the mRNA (Figure 2A) and protein levels in the brains of Aβo-treated mice compared to mock-treated controls (Figure 2C and E). Similarly, in the brains of 3xTg-AD mice, Mertk transcription (Figure 2B) and protein expression (Figure 2D and F) were significantly upregulated. Immunofluorescence analysis of Mertk expression in microglia of Aβo-treated mice demonstrated a significant increase (Figure 2G and I), with predominant localization in microglia rather than in astrocyte and neuron (Figure S3). Similarly, elevated Mertk expression was observed in the microglia of 3xTg-AD mice compared to WT mice (Figure 2H and J).

To assess morphological changes in microglia in AD, we conducted immunofluorescence staining using the microglial marker IBA1 in Aβo-treated and mock-treated mice. Microglia in the hippocampus of Aβo-treated mice displayed a greater number of intersections, more complex cellular processes, and enlarged soma compared to those in mock-treated mice (Figure 2K). Similar increases and morphological changes were noted in the microglia of 4-month-old 3xTg-AD mice (Figure 2L and Figure S2B). These results suggest that, in the early stage of AD, Aβo-induced changes enhance microglial surveillance and phagocytic functions in the surrounding environment. Given the role of microglia in synaptic engulfment, we next evaluated their phagocytic activity on synaptic components under Aβo treatment. Our results showed a marked increase in the volume of PSD95^+^ structures within microglia of Aβo-treated mice (Figure 2M-N). Similarly, in 4-month-old 3xTg-AD mice, significantly increased PSD95^+^ synaptic components were exhibited in microglia (Figure 2O-P). These findings suggest that synaptic phagocytosis by microglia is enhanced during the early stage of AD, in the presence of Aβo without Aβ plaque deposition.

**Figure 3:**
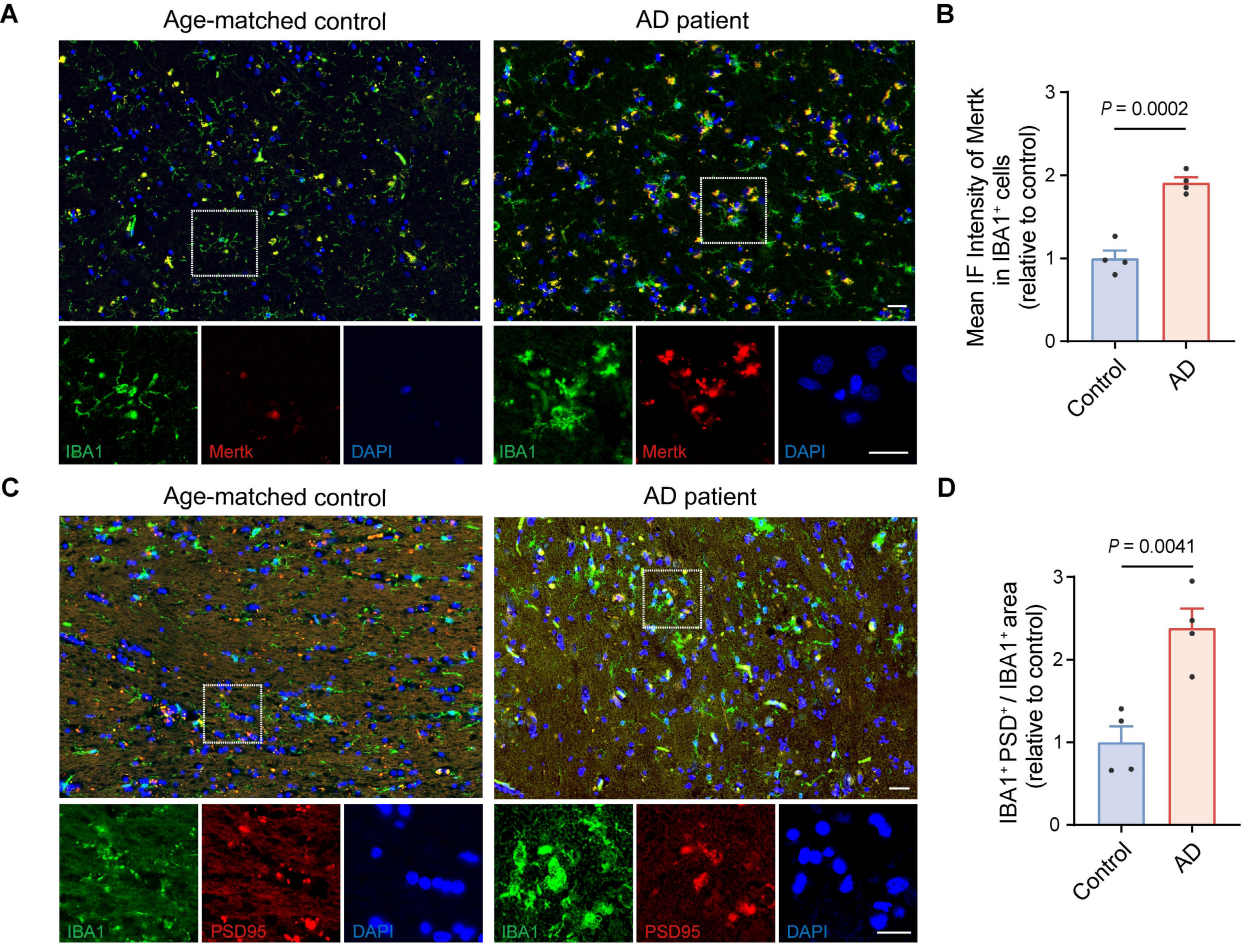
**Upregulation of Mertk in microglia accompanied by excessive synaptic engulfment in the AD brain.** (**A, B**) Immunostaining of Mertk (red), IBA1 (green) and DAPI (blue) in the hippocampus of AD patients and age-matched controls. Scale bar, 25 μm. (**C, D**) Immunostaining of PSD95 (red), IBA1 (green) and DAPI (blue) in the hippocampus of AD patients and age-matched controls. Scale bar, 25 μm. *n* = 4 cases/group; 4-6 fields per case were analysed using two-tailed unpaired *t*-test. Data are presented as means ± SEM.

Mertk has previously been reported to be abundantly expressed in microglia in normal human 48. Next, we performed Mertk immunoreactivity in microglia from AD patients and age-matched control subjects. Immunofluorescence microscopy of human hippocampal tissue confirmed that Mertk co-localized with the microglial marker IBA1 (Figure 3A-B). Mertk expression on microglia in AD patients was significantly increased—by almost twofold (*P* < 0.001)—compared with age-matched controls (Figure 3A-B), suggesting that Mertk plays a key pathogenic role in the development of AD. Simultaneously, we observed a nearly 2.5-fold increase in the co-localisation of PSD95 and IBA1 in the hippocampus of AD patients compared with age-matched controls (*P* < 0.01), indicating excessive engulfment of synaptic elements by microglia in AD (Figure 3C-D). Furthermore, we examined publicly available datasets related to AD research 49. Cognitive function in AD patients was assessed using the clinical dementia rating (CDR) scale 50, the Consortium to Establish a Registry for Alzheimer's Disease (CERAD) score 51, and Braak staging 52 to classify clinical severity. These analyses revealed a correlation between Mertk expression in the brains of AD patients and both the severity of cognitive impairment and clinical stage, compared with non-AD cohorts (Figure S4A-C). In conclusion, these results suggest that Mertk may be involved in disease progression during the early stage of AD.

### Mertk promotes synaptic defects and cognitive impairment in AD mice

**Figure 4:**
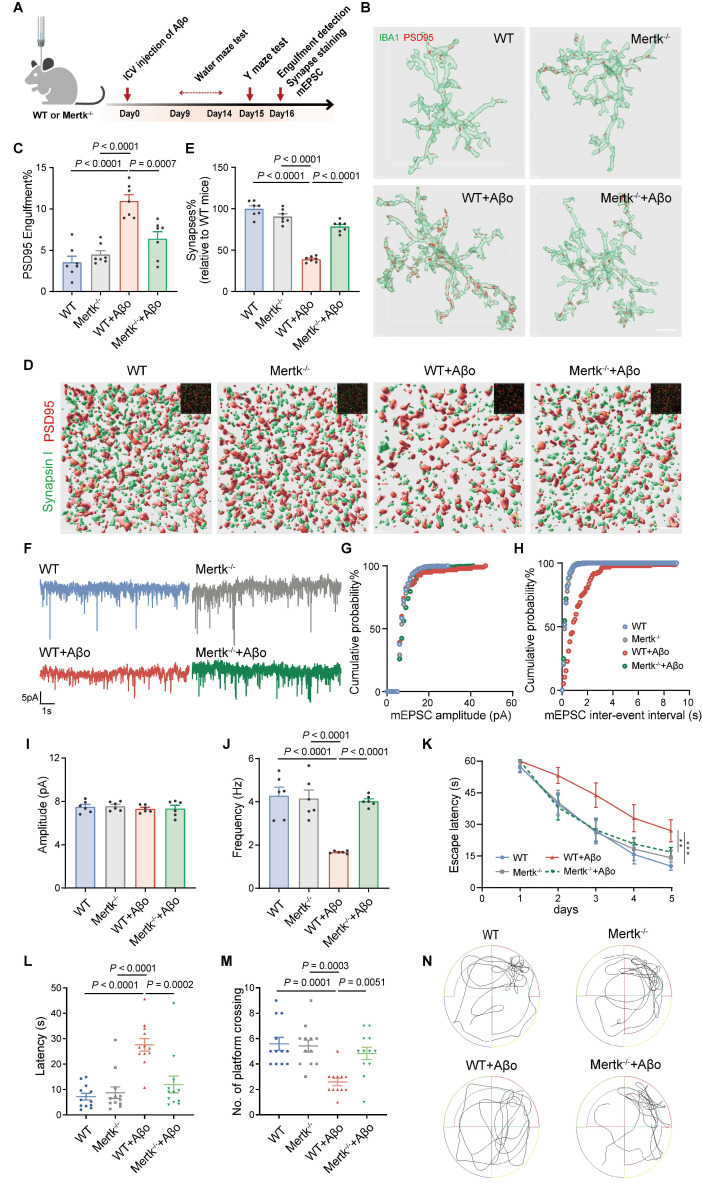
** Mertk deficiency mitigates Aβo-induced synapse loss by attenuating microglia-mediated synaptic elimination and alleviating behavioral deficits.** (**A**) Schematic of the experimental design. Y-maze and Morris water maze tests were conducted in different animal models. ICV: intracerebroventricular; mEPSC: miniature excitatory postsynaptic current. (**B, C**) Three-dimensional reconstructions and surface renderings show PSD95^+^ puncta within IBA1-positive microglia in the hippocampus. Scale bar, 10 μm. *n* = 7 mice/group; 6-8 microglia per mouse were analysed using one-way ANOVA with Dunnett's multiple comparisons test. (**D, E**) Three-dimensional reconstructions and surface renderings of synaptic puncta labeled with Synapsin I (presynaptic marker, green) and PSD95 (postsynaptic marker, red) in the hippocampus of the four groups. Synaptic numbers were determined by counting colocalized pre- and postsynaptic puncta (yellow). The inset in the upper right corner shows the fluorescent stain image before rendering. Scale bar, 5 μm. *n* = 7 mice/group; 8-10 fields per mouse were analysed using one-way ANOVA with Dunnett's multiple comparisons test. (**F**) Representative traces of mEPSCs recorded from the four groups. (**G, H**) Cumulative probability distributions of mEPSC amplitudes (G) and inter-event intervals (H) in the four groups. (**I, J**) Histograms of average mEPSC amplitude (I) and frequency (J) in the four groups (*n* = 10-12 cells/group from 6 mice; one-way ANOVA with Dunnett's multiple comparisons test). (**K**) Line chart of Morris water maze escape latency over five training days in the four groups (*n* = 12 mice/group; two-way repeated measures ANOVA with Tukey's multiple comparisons test). (**L, M**) Graph of the time spent to reach original platform location (L) and the number of platform crossings (M) during Morris water maze spatial probe trial (*n* = 12 mice/group; one-way ANOVA with Dunnett's multiple comparisons test). (**N**) Representative trajectory of the four groups during the spatial probe trial. Data are presented as means ± SEM. **P < 0.01, ***P < 0.001.

To investigate the role of microglial Mertk in synaptopathy during the early stage of AD, Aβo-induced AD models were generated in Mertk^-/-^ and WT mice via ICV injection of Aβo (Figure 4A). Microglial phagocytosis of synapses was assessed by measuring the volumes of PSD95^+^ structures within microglia. PSD95^+^ structures increased in Aβo-treated WT mice compared to mock-treated WT controls, whereas it was reduced in Aβo-treated Mertk^-/-^ mice compared to Aβo-treated WT mice. No significant differences were observed between untreated WT and Mertk^-/-^ mice, nor between Aβo- and mock-treated Mertk^-/-^ mice (Figure 4B-C). Double staining for PSD95 and Synapsin I revealed a reduction in synaptic density in the hippocampus of Aβo-treated WT mice compared to mock-treated WT controls, which was restored in synaptic density in Mertk^-/-^ mice (Figure 4D-E). Depletion of Mertk also increased the frequency, but not the amplitude, of miniature excitatory postsynaptic currents (mEPSCs) in CA3 neurons following Aβo treatment, consistent with enhanced synaptic preservation (Figure 4F-J).

Spatial learning and memory, assessed via the Morris water maze, showed that Aβo-treated WT mice had longer escape latencies and fewer platform crossings compared to mock-treated controls (Figure 4K-N). In contrast, Aβo-treated Mertk^-/-^ mice exhibited significantly shorter escape latencies and more platform crossings compared to Aβo-treated WT mice, suggesting that Mertk deficiency effectively reverses cognitive deficits. Y maze testing further showed a reduction in spontaneous alternations in Aβo-treated WT mice, which was restored in Mertk^-/-^ mice (Figure S5A-B). No significant differences were observed between mock-treated WT and Mertk^-/-^ mice. In summary, Mertk deficiency mitigates early synaptic loss and prevents Aβ-induced cognitive impairments in AD.

Additionally, it has been reported that aged mice exhibit increased Aβ42/Aβ40 ratios and tau aggregation 53, 54. Under such circumstances, we intervened in Mertk signaling in aged WT mice (12 months old) to explore the involvement of the Mertk pathway in AD pathology, such as Aβ levels and tau protein phosphorylation. We found no significant changes in the levels of Aβ1-42 or Aβ1-40 in the brains of 12-month-old Mertk^-/-^ mice compared with age-matched WT mice (Figure S6A). In contrast, phosphorylated tau levels were reduced by nearly 40% in Mertk^-/-^ mice compared with age-matched WT mice (*P* < 0.01), while total tau protein levels remained unchanged (Figure S6B-C).

**Figure 5:**
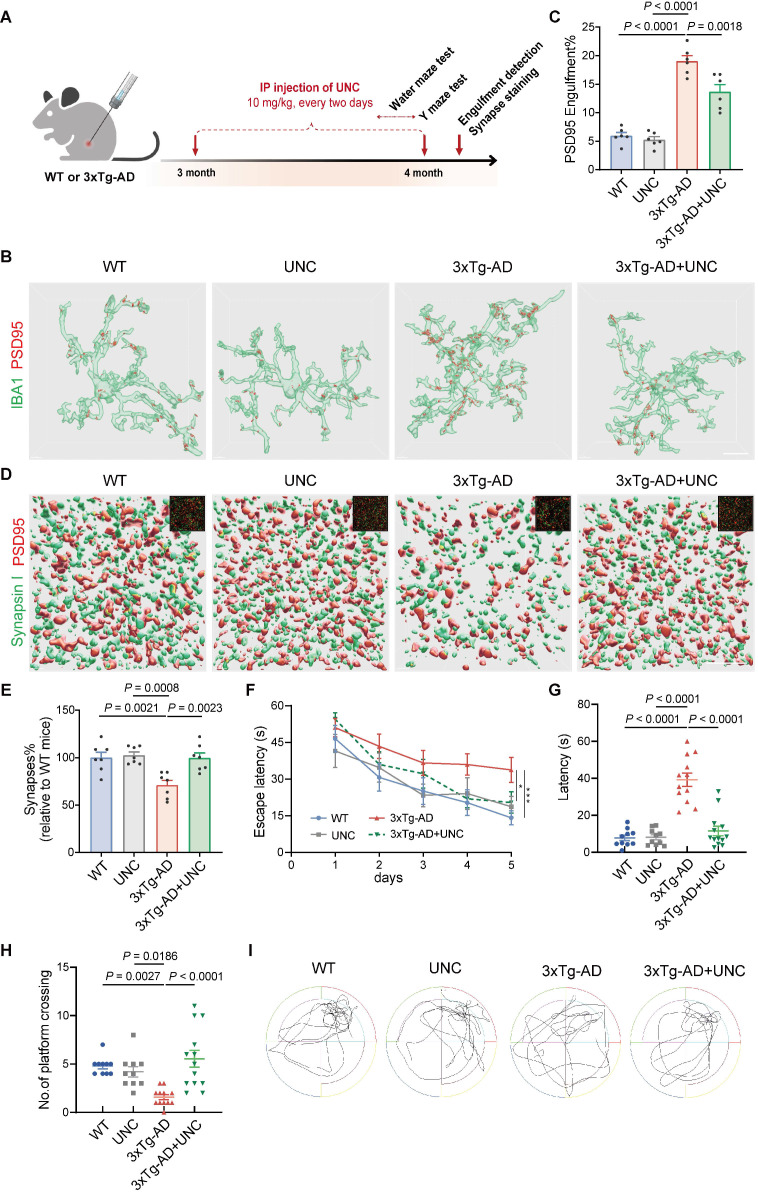
** Inhibiting Mertk reduces microglial engulfment of synapses and alleviates cognitive impairment in 3xTg-AD mice.** (**A**) Experimental schematic showing treatment and testing timeline. Y-maze and Morris water maze tests were conducted in different animal models. IP: intraperitoneal injection; UNC: UNC2250. (**B, C**) Three-dimensional reconstruction and rendering of PSD95^+^ puncta within IBA1-positive microglia in the hippocampus. Scale bar, 10 μm. *n* = 6 mice/group; 6-8 microglia per mouse were analysed using one-way ANOVA with Dunnett's multiple comparisons test. (**D, E**) Three-dimensional reconstructions and surface renderings of synaptic puncta labeled with Synapsin I (presynaptic marker, green) and PSD95 (postsynaptic marker, red) in the hippocampus. Synaptic numbers were determined by counting colocalized pre- and postsynaptic puncta (yellow). The inset in the upper right corner shows the fluorescent stain image before rendering. Scale bar, 5 μm. *n* = 7 mice/group; 8-10 fields per mouse were analysed using one-way ANOVA with Dunnett's multiple comparisons test. (**F**) Line chart of Morris water maze escape latency over five training days in the four groups (*n* = 10, 10, 12, 13 mice/group; two-way repeated measures ANOVA with Tukey's multiple comparisons test). (**G, H**) Graph of the time spent to reach original platform location (G) and the number of platform crossings (H) during Morris water maze spatial probe trial (*n* = 10, 10, 12, 13 mice/group; one-way ANOVA with Dunnett's multiple comparisons test). (**I**) Representative trajectory of the four groups during the spatial probe trial. Data are presented as means ± SEM. *P < 0.05, ***P < 0.001.

Other experiments to evaluate the effects of Mertk were conducted in 3xTg-AD mice (Figure 5A). UNC2250, the Mertk-selective antagonist, was administered via intraperitoneal (IP) injection for 1 month. Compared to WT mice, 3xTg-AD mice exhibited a significant increase in PSD95^+^ synaptic components within microglia (Figure 5B-C). However, UNC2250 treatment effectively reduced PSD95^+^ structures in the microglia of 3xTg-AD mice. There were no significant differences in PSD95^+^ structure volumes between mock- and UNC2250-treated WT mice or between UNC2250-treated WT and 3xTg-AD mice. UNC2250 treatment also reversed synaptic loss in the hippocampus of 3xTg-AD mice. Synaptic density, assessed through colocalized PSD95 and Synapsin I puncta, was significantly reduced in 3xTg-AD mice compared to WT mice (Figure 5D-E). In contrast, UNC2250 treatment increased synaptic density in 3xTg-AD mice. No significant differences in synaptic density were observed between mock- and UNC2250-treated WT mice, or between UNC2250-treated WT and 3xTg-AD mice.

Behavioral tests, including the Morris water maze and Y Maze, revealed that 3xTg-AD mice exhibited significant deficits in spatial learning and memory compared to WT mice (Figure 5F-I and Figure S5C-D). However, UNC2250 treatment significantly alleviated these deficits in 3xTg-AD mice. No significant differences in behavioral performance were observed between mock- and UNC2250-treated WT mice, or between UNC2250-treated WT and UNC2250-treated 3xTg-AD mice. In conclusion, inhibition of Mertk rescues early synaptic loss and ameliorates cognitive impairment in 3xTg-AD mice.

### Decreased engulfment of synaptic structures in Mertk-deficient microglia under Aβo challenge

**Figure 6:**
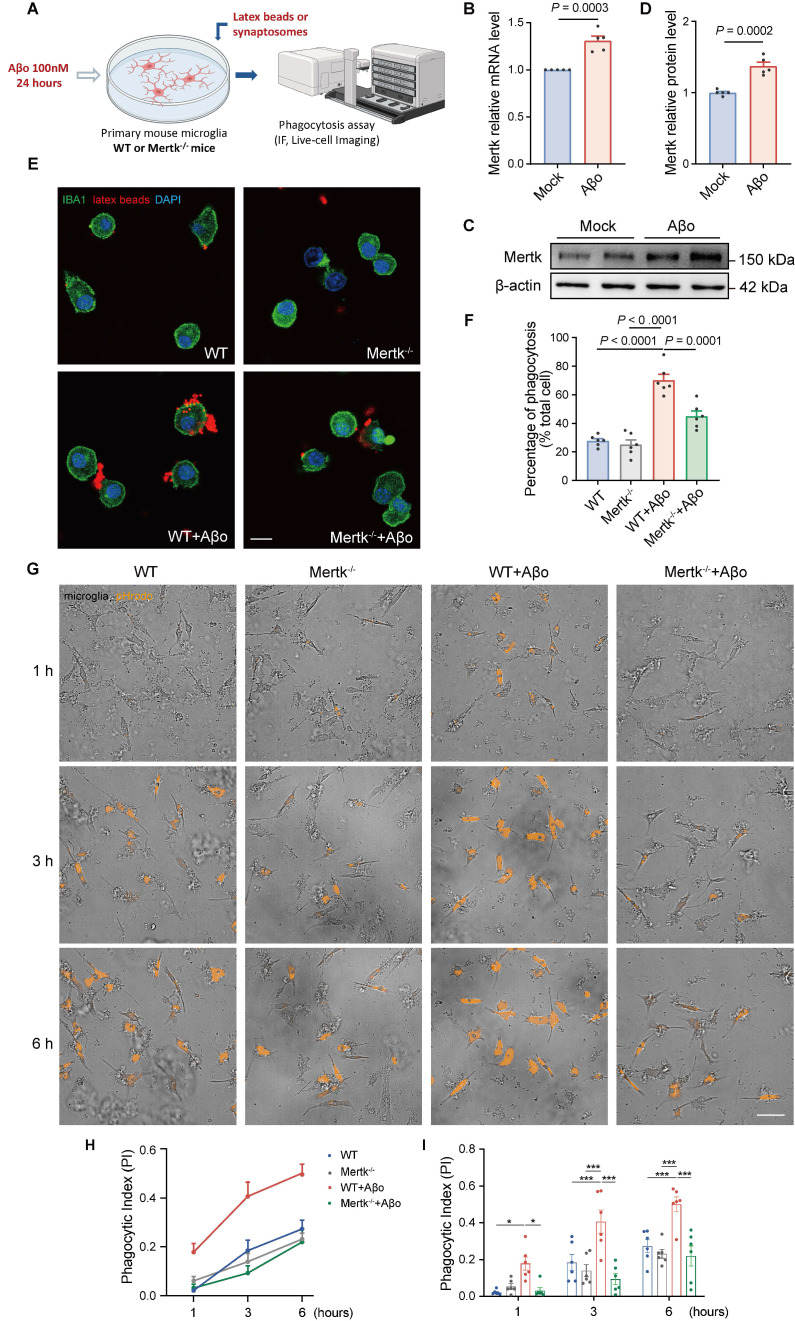
** Mertk mediates microglial phagocytosis induced by Aβo *in vitro*.** (**A**) Schematic representation of the experimental procedures. IF: immunofluorescence. (**B-D**) Mertk expression in primary microglia at the mRNA (B) and protein levels (C), with quantification shown in (D). *n* = 5 independent experiments; two-tailed unpaired *t*-test. (**E**) Representative immunofluorescence images showing latex beads (red) engulfed by IBA1-positive primary microglia (green). Scale bar, 10 μm. (**F**) Quantification of microglial phagocytosis, represented as the ratio of the total area of fluorescent signals to the total number of phagocytic cells. *n* = 6 wells; average of 6-8 fields from each well was analysed using one-way ANOVA with Dunnett's multiple comparisons test. (**G**) High-Content Live-Cell Imaging shows phagocytosed pHrodo red-conjugated synaptosomes (orange) engulfed by primary microglia at representative time points. Scale bar, 50 μm. (**H**) Phagocytic index (PI) of primary microglia over time. (**I**) Quantification of phagocytic index in different groups at representative time points. *n* = 6 wells; average of 6-8 fields from each well was analysed using two-way ANOVA with Tukey's multiple comparisons test. Data are presented as means ± SEM. *P < 0.05, ***P < 0.001.

To verify microglial responses to Aβo treatment *in vitro*, we stimulated primary microglia from WT mice with Aβo (100 nM) for 24 h and assessed Mertk expression (Figure 6A). Both Mertk mRNA and protein levels were upregulated after Aβo stimulation compared to mock-treated controls (Figure 6B-D). Next, we examined the impact of Mertk on microglial phagocytosis in Aβo-treated primary microglia from WT and Mertk^-/-^ mice. Aβo stimulation significantly increased the uptake of fluorescent beads by microglia compared to mock treatment (Figure 6E-F). However, Mertk depletion reduced the uptake ratio of fluorescent beads and suppressed phagocytosis. These results suggest that Mertk mediates the phagocytosis of microglia under Aβo stimulation.

To further explore phagocytosis of synapses by microglia, synaptosomes were extracted from WT mouse brains and labeled with pHrodo dye. Then, pHrodo-conjugated synaptosomes were co-cultured with microglia, which were pretreated with or without Aβo for 24 h. Using High-Content Live-Cell Imaging, we recorded microglial phagocytic activity in real time over 6 h (Figure 6G and Video S1-4). No significant difference in phagocytic index (PI) was observed between untreated WT and Mertk^-/-^ microglia (Figure 6H-I and Figure S7A-B). However, both phagocytic index and pHrodo intensity were increased in Aβo-treated microglia compared to mock treatment. In contrast, Mertk^-/-^ microglia exhibited a reduction in phagocytic index under Aβo stimulation (Figure 6H-I and Figure S7A-B). These results suggest that Mertk deficiency prevents microglial phagocytosis of synapses in response to Aβo challenge.

### PPARγ regulates Mertk-mediated microglial phagocytosis induced by Aβo

**Figure 7:**
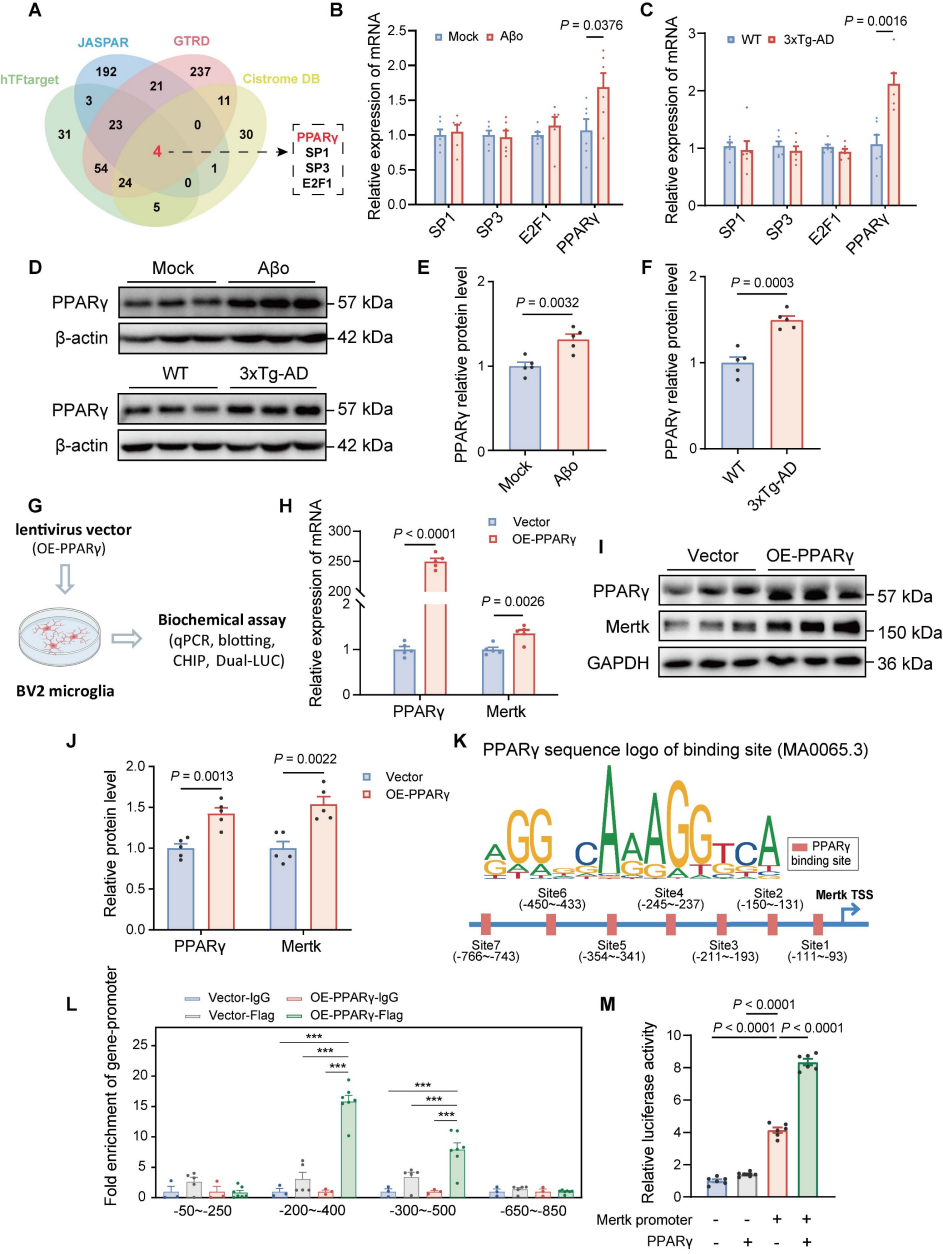
** PPARγ is upregulated in the early stage of AD mice and promotes Mertk transcription.** (**A**) Bioinformatics analysis identified PPARγ as a potential transcription factor driving Mertk expression. (**B-F**) PPARγ mRNA levels (B, C) and protein levels (D-F) are upregulated in Aβo-treated mice and in 4-month-old 3xTg-AD mice. *n* = 5 mice/group; two-tailed unpaired *t*-test. (**G**) Schematic representation of the experimental procedures. OE, overexpression. (**H-J**) PPARγ and Mertk mRNA levels (H) and protein levels (I, J) in a stable BV2 cell overexpressing PPARγ. *n* = 5 independent experiments; two-tailed unpaired *t*-test. (**K**) PPARγ binding site sequences predicted using the JASPAR database. The size of the bases reflects the probability of occurrence at each position. The blue line represents the Mertk promoter sequence, with arrows indicating the transcription start site (TSS). The red boxes denote potential PPARγ binding sites, annotated with their distances from the TSS. (**L**) ChIP experiments validated the promoter regions bound by PPARγ. *n* = 3 independent experiments; one-way ANOVA with Dunnett's multiple comparisons test. (**M**) Dual-luciferase reporter assays confirmed the interaction between PPARγ and the Mertk promoter. *n* = 6 independent experiments; one-way ANOVA with Dunnett's multiple comparisons test. Data are presented as means ± SEM. ***P < 0.001.

Both *in vivo* and *in vitro* studies demonstrate that Aβo stimulation enhances Mertk gene transcription and protein expression. To elucidate the mechanisms underlying Mertk upregulation in microglia induced by Aβo, we searched the hTFtarget, JASPAR, GTRD, and Cistrome DB databases for transcription factors that may activate Mertk transcription. This search identified four candidate transcription factors: SP1, SP3, E2F1, and PPARγ (Figure 7A). In comparison to the control group, the mRNA levels of SP1, SP3, and E2F1 in the brain tissue of Aβo and 3xTg-AD mice showed no substantial differences, but PPARγ was significantly upregulated (Figure 7B-C). Thus, PPARγ emerged as the potential transcription factor governing Mertk and was selected for further investigation. Western blot analyses revealed a trend toward increased PPARγ protein levels both in Aβo-treated and 3xTg-AD mice compared to normal mice (Figure 7D-F).

To investigate further, we established a stable BV2 microglial cell line overexpressing PPARγ using lentiviral infection (Figure 7G). Notably, PPARγ overexpression resulted in a significant increase both in Mertk mRNA and protein levels compared to vector-treated BV2 cells (Figure 7H-J). To further elucidate PPARγ's role as a transcription factor regulating Mertk, we predicted PPARγ binding sites using JASPAR. Comparative analysis of the identified binding sequences with the Mertk promoter revealed seven potential PPARγ binding sites (Figure 7K). Chromatin immunoprecipitation (ChIP) assays and dual-luciferase reporter assays confirmed that PPARγ can bind to the Mertk promoter in the upstream region (-200 to -500 bp) relative to the transcription start site, facilitating transcription (Figure 7L-M).

**Figure 8:**
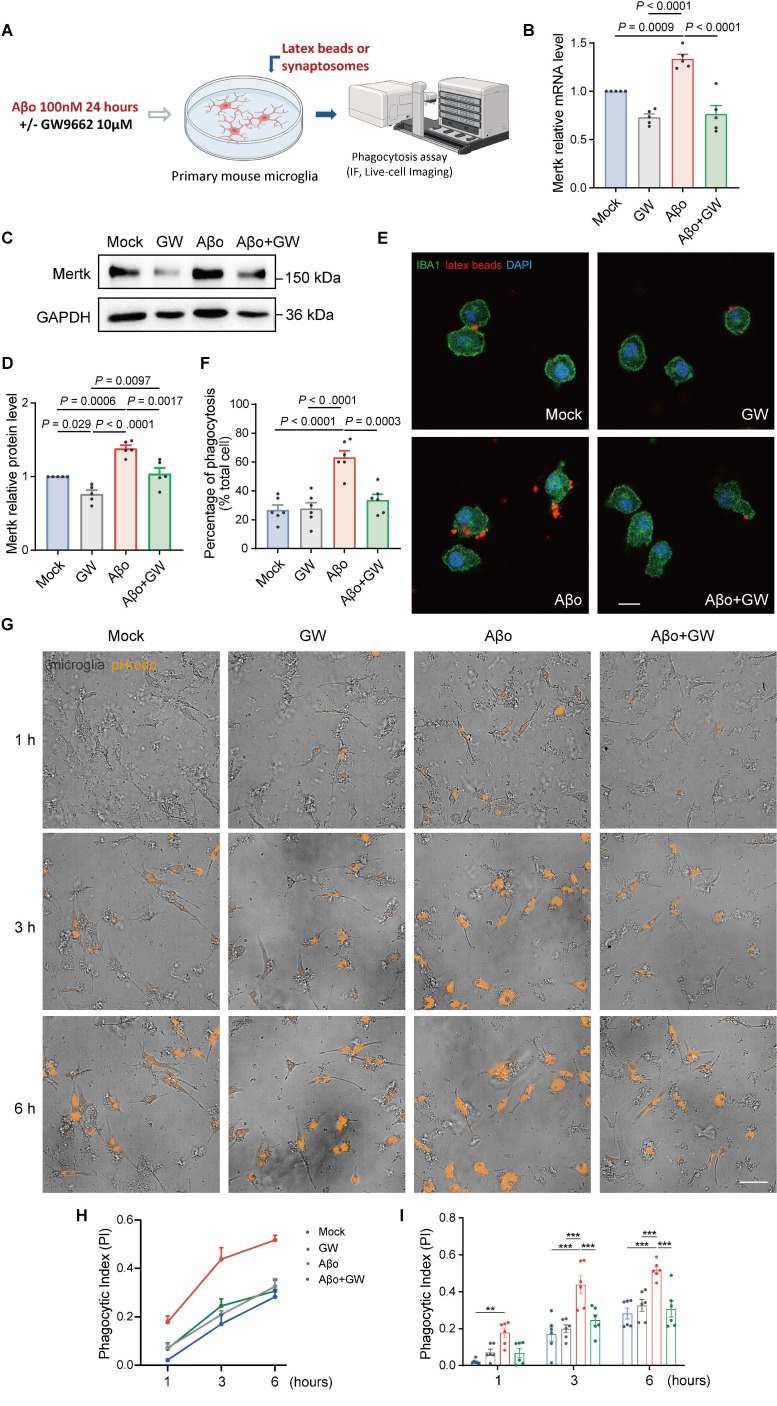
** PPARγ participates in microglial phagocytosis by regulating Mertk under Aβo stimulation.** (**A**) Schematic representation of the experimental procedures. IF: immunofluorescence. (**B-D**) Mertk mRNA levels (B) and protein levels (C, D) showed downregulation following GW9662 treatment in Aβo-induced primary cultured microglia. *n* = 5 independent experiments; One-way ANOVA with Dunnett's multiple comparisons test. (**E**) Representative immunofluorescence images show latex beads (red) engulfed by IBA1-positive primary microglia (green). Scale bar, 10 μm. (**F**) Quantification of microglial phagocytosis was presented as the ratio of the total area of fluorescent signals to the total number of phagocytic cells. *n* = 6 wells; average of 6-8 fields from each well was analysed using one-way ANOVA with Dunnett's multiple comparisons test. (**G**) High-Content Live-Cell Imaging reveals phagocytosed synaptosomes (orange) engulfed by primary microglia at representative time points. Scale bar, 50 μm. (**H**) Phagocytic index (PI) of primary microglia over time. (**I**) Quantification of phagocytic index in different groups at representative time points. *n* = 6 wells; average of 6-8 fields from each well was analysed using two-way ANOVA with Tukey's multiple comparisons test. Data are presented as means ± SEM. **P < 0.01, ***P < 0.001.

To further investigate whether PPARγ takes part in Mertk-mediated synaptic pruning, primary microglia were stimulated with Aβo and concurrently treated with GW9662 (a selective PPARγ antagonist) for 24 h (Figure 8A). Mertk mRNA and protein levels in microglia were upregulated within 24 h following Aβo stimulation, compared to mock-treated controls. As expected, GW9662 significantly downregulated Mertk expression at both mRNA level and protein level following Aβo stimulation (Figure 8B-D).

Next, we examined the impact of the PPARγ pathway on microglial phagocytosis. Aβo significantly increased the uptake of fluorescent beads in primary microglia compared to mock treatment, whereas GW9662 effectively attenuated the fluorescent beads uptake ratio under Aβo stimulation (Figure 8E-F). This indicates that the PPARγ inhibitor suppresses microglial phagocytic activity during Aβo challenge.

Furthermore, to assess the effect of PPARγ inhibition on synaptic phagocytosis, microglia were co-incubated with Aβo and GW9662 for 24 h, after which they were co-cultured with pHrodo-conjugated synaptosomes. Phagocytosis of synaptosomes was then evaluated *via* High-Content Live-Cell Imaging in real time over 6 h (Figure 8G and Video S5-8). Both the phagocytic index and pHrodo intensity showed no significant differences between mock and GW9662-treated groups (Figure 8H-I and Figure S7C-D). As time progresses, there were increases in both the phagocytic index and pHrodo intensity in all four groups. As expected, an increase in phagocytic index was observed in microglia following Aβo treatment compared to mock treatment. Interestingly, Aβo-induced pHrodo index in microglia was reduced by GW9662 at the third and sixth hours, and a decreasing trend was explained in the first hour (*P* = 0.053). Similar changes in pHrodo intensity were observed in Aβo-induced microglia in the presence or absence of GW9662 (Figure S7C-D).

Meanwhile, we also evaluated the effects of PPARγ inhibition on spatial learning and memory in 3xTg-AD mice. Significant impairments were observed in the Morris water maze and Y-maze tests in 3xTg-AD mice compared to WT controls (Figure S8A-G). Administration of GW9662, however, effectively alleviated these deficits in 3xTg-AD mice. Collectively, these findings suggest that PPARγ regulates Mertk, contributing to Aβo-induced microglial phagocytosis and behavioral impairments.

## Discussion

In this study, we found that early excessive synaptic pruning by microglia occurs in AD, when only Aβo are present, prior to plaque formation. Aβo-induced microglial phagocytosis is mediated by the Mertk receptor and regulated by the PPARγ pathway. Inhibition or knockout of Mertk reduces microglial synapse engulfment, prevents synaptic loss, and improves behavioral deficits in early stage AD mice.

We found that Mertk expression was significantly upregulated in the brains of AD patients, suggesting its involvement in AD progression, potentially including excessive synaptic pruning and/or internalisation of synaptic components by microglia. These findings underscore that Mertk plays a key pathogenic role in the development of AD, as it functions as a phagocytic receptor in both physiological and pathological pruning 42, 44, and also provide a basis for considering Mertk as a potential therapeutic target in AD management. Furthermore, our results indicate a significant association between Mertk expression and cognitive function in AD. While there is no definitive evidence of a direct causal link between Mertk and AD, prior studies have proposed Mertk as a key risk factor contributing to AD pathogenesis 55.

Our observations are supported by others, showing enhanced Mertk expression in microglia surrounding Aβ plaques in aged AD mice 46. In the late stages of AD, Mertk interacts with the phosphatidylserine (PtdSer)-rich dystrophic membranes of plaques, facilitating microglial detection, phagocytosis, and compaction of Aβ plaques. The formation of dense-core plaques prevents the spread of toxic pre-plaque Aβo across the brain, thus alleviating cognitive impairments in fear acquisition 46. However, in our current study, prior to Aβ plaque formation, Mertk mediates microglial phagocytosis of synapses induced by Aβ oligomers, leading to early synaptic loss and behavioural deficits in AD. Our findings align with others, showing that microglial Mertk phagocytic pathways specifically target and eliminate PtdSer-exposed synapses 44, suggesting that Mertk mediates phagocytosis of PtdSer-exposed synapses by microglia at early AD stages. Based on these findings, it is plausible that Mertk-mediated phagocytosis by microglia occurs through its ligand GAS6 at both early and late AD stages by recognising PtdSer, leading to engulfment of PtdSer-containing structures. The difference lies in the identity of PtdSer-labelled substrates at each stage, meaning the material phagocytosed by microglia varies between early and late AD. Furthermore, studies on APP/PS1 mice at different ages indicate that Mertk mRNA levels increase initially and then decrease 56, suggesting a complex role of Mertk in AD and highlighting the importance of exploring its involvement in the early stage of the disease.

The establishment of an AD model using ICV injection of Aβo has been widely adopted in studies investigating AD pathogenesis 28, 57-59. The current injection model, which is well-documented, involves lateral ventricle injection of Aβo 60. This model exhibits AD-like behavioural alterations, including learning and memory impairment, neuronal dysfunction (such as synaptic protein loss, dendritic spine reduction, and electrophysiological defects) 61, 62, as well as microglial activation with impaired phagocytic function 28. Our experimental results also show that this AD model exhibits increased microglial phagocytosis of synapses compared to mock-treated controls. Additionally, synapse loss was observed, alongside impairments in spatial learning and memory demonstrated in the Morris water maze and Y maze tests. These findings provide further validation of the AD model. Furthermore, our research aims to investigate the abnormal phagocytosis of synapses by microglia under Aβo exposure prior to plaque formation. Therefore, the Aβo lateral ventricle injection model is appropriate for our research objectives. The injection model used in this study offers a more precise and controlled approach to AD induction, whereas transgenic AD mouse models involving mutations in APP, PS1, and other genes often involve multiple pathological processes beyond Aβ accumulation, resulting in a more complex disease state. In addition to demonstrating upregulation of the Mertk pathway via single-cell sequencing of Aβo-injected AD mice, we further validated the underlying mechanism *in vivo* using both the Aβo injection model and 4-month-old 3xTg-AD mice.

After two-week Aβo challenge, single-cell sequencing identified ten distinct cell types, with a notable increase in microglia. This is consistent with KEGG analyses, which revealed enhanced phagocytosis and efferocytosis functions in microglia, emphasizing their role in the early stage of AD development. Our findings align with previous studies suggesting that soluble Aβ triggers microglial activation and subsequently leads to phagocytosis and clearance of Aβ 63.

Microglia normally function protectively in the brain, acting as "housekeepers"—phagocytes that maintain tissue homeostasis by clearing extracellular Aβ, thereby preventing AD 64. However, gradually increasing toxic Aβ activates microglia, which then release inflammatory mediators, further driving AD progression in susceptible individuals 65. Consequently, these activated microglia may promote inflammation and/or disrupt homeostasis 66, leading to reduced Aβ clearance in affected regions. Synapses usually send "don't eat me" signals, such as *via* the CD47-SIRPα pathway, to prevent abnormal microglial engulfment under normal physiological conditions 31. This aligns with other reports showing that in the early stage of AD, elevated Aβ can hyperactivate the C1q/C3 complement system, substantially increasing "eat me" signals and disrupting the balance between "eat me" and "don't eat me" cues 29, 32. This shift results in a higher number of synapses expressing "eat me" signals, making them more susceptible to recognition and engulfment by abnormally activated microglia in AD.

Our data show enhanced morphological changes in microglia from Aβo-induced AD mice, suggesting an increase in microglial monitoring and phagocytic capabilities during AD development. This is consistent with previous studies showing that activated microglia adopt an enlarged soma with finer processes 67. Additionally, we observed excessive microglial phagocytosis of synapses in Aβo-induced AD mice, resulting in synaptic loss, decreased mEPSC frequency, and behavioral deficits. These findings suggest that microglia not only clear Aβ but also excessively engulf synapses in early stage of AD, promoting disease progression. These findings were further confirmed in 4-month-old 3xTg-AD mice, which exhibit Aβo immunoreactivity but lack plaque formation. Our results in two AD models were consistent with reports showing inappropriate activation of the complement-dependent pathway and microglia during synaptic pruning in AD 28.

To investigate microglial subpopulations involved in synaptic pruning under Aβo stimulation, we clustered microglia into six groups. Cluster 3, associated with phagocytosis, showed upregulation of the GAS6-Mertk pathway. These findings were further confirmed in both Aβo-treated and 3xTg-AD mice, where Mertk expression was increased at both mRNA and protein levels.

Our data support the role of Mertk in AD, with predominant localization in microglia rather than astrocytes. Additionally, there is a close correlation between Mertk expression and microglial synapse engulfment from the early stage of AD, particularly in the presence of Aβo without Aβ plaque deposition. Our findings illustrate that Mertk plays an important role in early AD progression via microglial-mediated synaptic engulfment, which consequently impairs cognitive function. Moreover, the relationship between Mertk expression in astrocytes and AD involves extracellular matrix (ECM) remodelling, which promotes Aβ plaque phagocytosis by astrocytes, through activation of astrocytic Mertk and enhanced astrocytic vesicle circulation 68. Interestingly, studies on astrocytes have shown that stress hormones induce astrocyte-mediated synapse elimination by triggering glucocorticoid receptor (GR)-driven Mertk expression during early life. This correlates with altered neural activity patterns and increased helpless behaviour in adulthood 43. Furthermore, previous research reported that Mertk protein is highly localized to developing astrocytes in the postnatal day 5 dorsal lateral geniculate nucleus (dLGN), and that Mertk depletion suppresses retinal synaptic pruning and leads to retinal degeneration 42. These findings suggest that Mertk-mediated astrocytes participate in synaptic pruning during neonatal brain development. Our current research, however, focuses on early synaptic damage in AD mice that are already adults with completed neural development. Specifically, we focus on the telencephalon—particularly the hippocampus—where microglia show enhanced synaptic engulfment. In contrast, during development under stress, astrocytes in the somatosensory cortex, rather than in the adult hippocampus, exhibit increased phagocytic activity, suggesting regional differences in stress-induced astrocytic responses 43. Therefore, the observed discrepancy between astrocytes and microglia may relate to differences in brain region and/or age, particularly regarding the roles of Mertk in the brain and retina.

To further confirm Mertk's contribution to the early stage of AD, we manipulated Aβo-induced and 3xTg-AD mouse models with Mertk knockout or inhibition before plaque formation, resulting in reduced microglial synaptic elimination and improved behavioral outcomes. These results verify Mertk as a key receptor in microglial synaptic elimination during the early stage of AD, where Aβo are present but plaques have not yet formed, and suggest that Mertk may be involved in the synaptic toxicity of Aβo. Although molecules like TREM2 34 and SIRPα 32 regulate microglia-mediated synaptic removal in AD progression, they are also involved in other microglial activities, including plaque phagocytosis 32, 69. These effects mainly occur in the middle to late stages of AD when pathological features are more pronounced. The differences between our findings and others highlight the variability in microglial states at different AD stages, with Mertk emerging as a novel mechanism for synaptic pruning earlier in the disease course. Literature reports suggest that C1q and Aβo co-activate the complement cascade, driving microglial synapse elimination via CR3 and contributing to early synaptic loss 28, 29. However, excessive or dysregulated complement activation can lead to overactive inflammation and further synaptic damage 24, 70, 71. In contrast, it has been reported that Mertk alleviates neuroinflammation in the brain 72, indicating that Mertk-mediated early synaptic pruning operates as a non-inflammatory mechanism in AD. This finding could provide potential therapeutic insights for AD treatment.

Our data show that 12-month-old Mertk^-/-^ mice exhibit approximately 40% lower phosphorylated tau levels than WT mice, with no change in total tau, suggesting a role for Mertk in AD development via tau phosphorylation and consequent cognitive decline. While no previous studies have linked Mertk to tau phosphorylation in AD, tau is phosphorylated by kinases such as GSK3β 73 and MAPK 74, and Mertk is closely associated with these pathways 75, 76, providing indirect support for our findings. Aβ levels did not differ between aged Mertk^-/-^ and WT mice, consistent with the low Aβ expression in aged non-AD mice 77, and no Aβ deposition was detected. The effects of long-term Mertk intervention on Aβ deposition and tau phosphorylation could be studied in the future.

*In vitro* validation of AD animal model observations revealed that Aβo induces Mertk expression in primary microglia, enhancing their phagocytic activity toward fluorescent beads and synaptosomes. Mertk depletion reduced this engulfment and protected synapses from excessive microglial activity. Our data align with studies indicating Mertk's role in microglial phagocytosis of plaques in later AD stages 46, though in the early stage, Aβo-triggered microglia excessively phagocytose synapses via Mertk.

Furthermore, we explored Mertk transcriptional regulation and identified PPARγ as a promising factor upregulated in Aβo-treated and 3xTg-AD mice, suggesting its potential involvement in AD development. Overexpression of PPARγ in microglial cell lines increased Mertk expression, and ChIP and Dual-LUC confirmed that PPARγ binds to the Mertk promoter, regulating its role in microglial phagocytosis. Our findings align with others showing PPARγ upregulation of Mertk expression in microglia 72, 78. We found that in Aβo-induced primary microglia, PPARγ activation enhanced Mertk transcription and synaptic phagocytosis, leading to synaptic damage and behavioral defect. This was confirmed by a PPARγ antagonist, which reduced microglial phagocytosis, suggesting PPARγ's role in synaptic loss and cognitive impairment. These findings contrast with studies on PPARγ agonists in AD, where chronic treatment with *pioglitazone*, a PPARγ agonist, reduces Aβ oligomer neurotoxicity by promoting Aβ fibril formation 79. Other agonists, like *pioglitazone* 80 and *rosiglitazone* 81, improve lipid metabolism and reduce inflammation, potentially benefiting AD pathology by enhancing neuronal survival. A small pilot study suggests that *rosiglitazone* treatment for 6 months may improve attention and memory in patients with MCI and AD 82, but results from large-scale trials have been inconsistent 83, highlighting the need for further investigation into PPARγ's role in AD. Compared to our data, these studies suggest that PPARγ may exert opposing effects in neurons and microglia at different AD stages. Thus, indiscriminate use of PPARγ agonists may exacerbate synaptic loss by increasing microglial phagocytosis, which may explain the inconsistent outcomes in clinical trials. Future research should focus on cell-targeted therapies and optimal treatment timing.

## Conclusions

**Figure 9:**
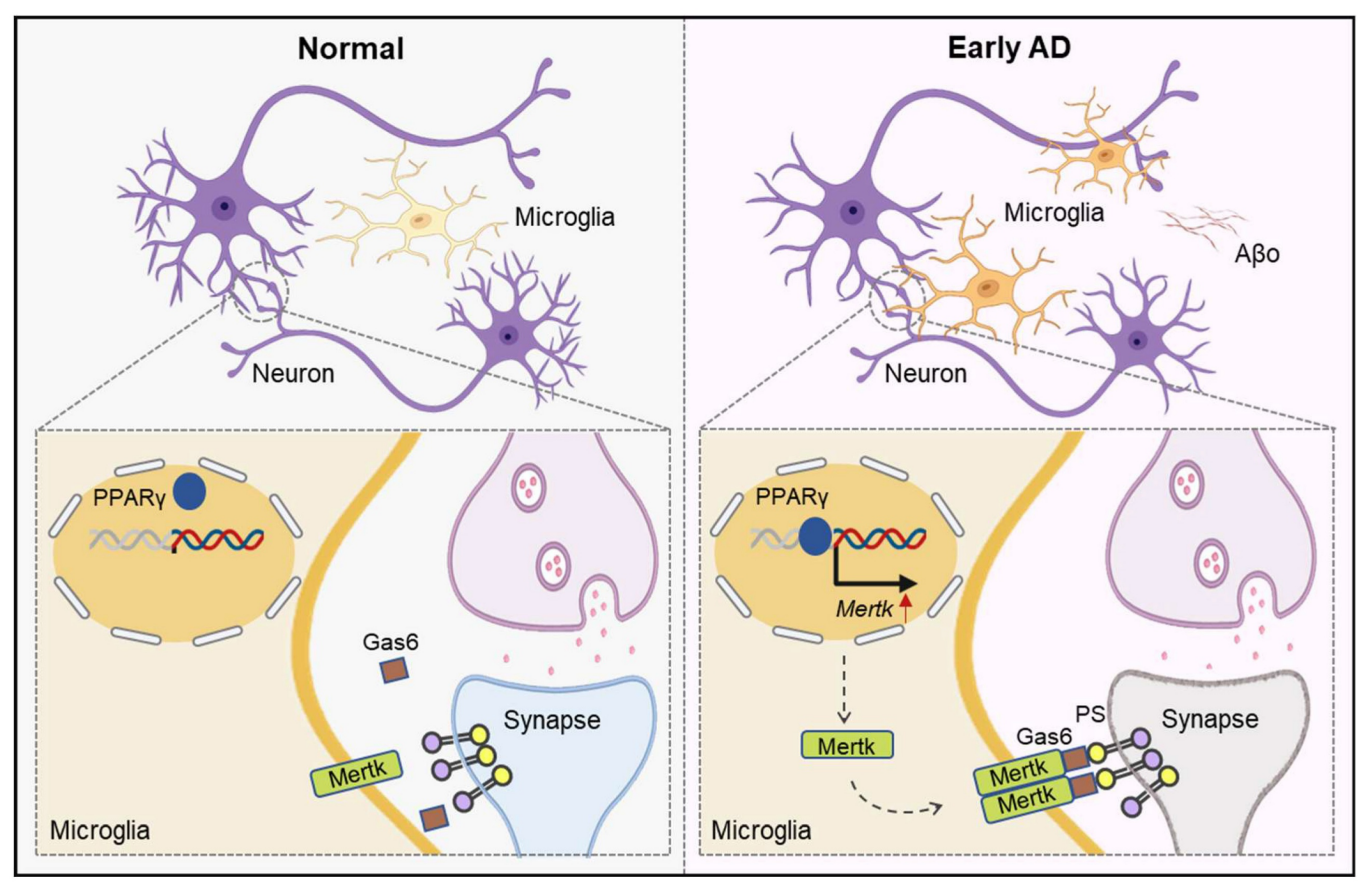
** Schematic illustration of the general mechanism.** In brief, Aβo stimulates PPAR-γ, enhancing Mertk transcription. Mertk drives excessive synaptic pruning by microglia, ultimately leading to early synaptic loss in AD.

In summary, our results demonstrate that prior to plaque formation, Aβo promote a microglial phagocytic phenotype in the early stage of AD, with Mertk mediating excessive synaptic engulfment by microglia, leading to cognitive deficits (Figure 9). Therefore, early intervention targeting Mertk may represent a potential therapeutic strategy to mitigate Aβo-induced, microglia-mediated early synaptic loss and behavioral impairments in AD.

## Materials and Methods

### Animals and procedures

Triple transgenic AD mice (3xTg-AD, Cat No. D00026) and age-matched Wild-type (WT) C57BL/6 J mice were purchased from Changzhou Cavens Model Animal Co.,Ltd (Changzhou, Jiangsu, China), and were randomly assigned simultaneously to two groups: the solvent-treated and the UNC2250-treated group. Starting at 3 months of age, mice were intraperitoneally injected with UNC2250 (10 mg/kg, MCE, USA) or the control solvent every other day for 1 month. The drug concentration was based on previously published literature 84. For PPARγ inhibition, mice were administered daily intraperitoneal injections of the PPARγ-selective antagonist GW9662 (1 mg/kg, Sigma-Aldrich, USA), for three weeks before the age of 4 months, with the dosage referenced in previously published literature 85.

Mertk knockout (Mertk^-/-^, Cat No.T012765) and Wild-type (WT) C57BL/6 J mice were purchased from GemPharmatech Co. Ltd (Nanjing, Jiangsu, China), and were randomly divided simultaneously into two groups: the mock-operated (mock) group and the intracerebroventricular (ICV) injection of Aβ1-42 oligomers (Aβo) group. The establishment of AD mouse model by injecting Aβ1-42 into the lateral ventricle has been adopted by many studies 57, 58. All the mice were maintained under constant environmental conditions in the Wuhan University Center for Animal Experiment with free access to food and water under a 12 h light/day cycle. All animal experimental protocols were approved by the Institutional Animal Care and Use Committee of Wuhan University and in accordance with institutional regulations.

To construct Mertk knockout mice 86, CRISPR/Cas9 was used to knockout exon 2 of the Mertk-201 transcript (ENSMUST00000014505.4), which contains a 409 bp coding sequence. Deletion of this region induces a frameshift mutation, disrupting protein function. The Mertk-202 transcript may remain unaffected. Heterozygous mice carrying Mertk deletion alleles were crossed to generate knockout mice, and their genotypes were confirmed by PCR using the primers listed in Table S1. The knockout status of Mertk was provided in Figure S9.

For Aβo treatment experiment, Aβo peptides were manufactured by Chinapeptides Co.,Ltd (> 95% purity) and the oligomers were prepared as described previously 58. Mice were anesthetized by isoflurane and placed in a stereotaxic frame, the bregma point of the skull was exposed. Aβo (400 pmol/5 μL/5 min/mouse) was stereotaxically infused bilaterally injected into the lateral ventricle (-2.3 mm dorsoventral (DV), -0.2 mm anteroposterior (AP), and ± 0.9 mm mediolateral (ML) from bregma) using a Hamilton syringe. After injection, the needle was left in place for 5 min and slowly withdrawn to prevent reflux of liquid. Two weeks later 59, the mice underwent behavioral tests, followed by euthanasia, and their brains were harvested for further analysis.

### Human brain tissue

Human brain hippocampal tissues from postmortem AD patients and age-matched, non-dementia control subjects were obtained from Institute of Forensic Medicine (Wuhan University, Wuhan, China). Case information is provided in Table S2. For post-mortem sample collection, we obtained the specimens with respect to the wishes of the deceased and their family, following all legal and ethical guidelines. All experiments were approved by the Medical Ethics Committee of Wuhan University.

### Primary microglial cultures and treatment

Primary microglia cultures were prepared as previously described 87. Briefly, brains were removed from WT or Mertk^-/-^ mice at postnatal day 1-3. After removal of the meninges and triturated brain tissue, the dissociated cells were cultured in DMEM/F12 media (Gibco, USA) supplemented with 10% FBS (Gibco, USA) and 1% penicillin/streptomycin (Biosharp, China) for 14 days. Finally, the matured microglia were harvested by shaking at 200 rpm for 2 h in a constant temperature shaker set to 37˚C. The dispersed cells were then collected, counted, seeded into plates, and allowed to rest overnight. For Mertk expression and phagocytosis assay, microglia were stimulated with 100 nM Aβo for 24 h before analysis. For the role of PPARγ in microglia phagocytosis assay, microglia were stimulated with 100 nM Aβo and co-cultured with PPARγ antagonist GW9662 (10 μM, Sigma-Aldrich, USA) for 24 h before analysis.

### BV2 microglia cultures and lentiviral transduction

The murine microglia cell line BV2 was obtained from the China Infrastructure of Cell Line Resources (Beijing, China) and cultured in 90% DMEM (Gibco, USA), 10% FBS (Gibco, USA) at 37℃ in a humidified atmosphere of 5% CO_2_. BV2 cells were seeded at a density of 12 × 10^4^cells/well on a 12-well plate format and the lentiviral vector transduction was performed 24 h after seeding the cells as described previously 88. BV2 cells were transfected with lentiviral vectors pLV2-3xFLAG-Puro (control) or pLV2-CMV-Pparg-3xFLAG-Puro (miaolingbio, China) in the presence of polybrene (5 μg/mL, MCE, USA). Stable cells were selected in the presence of puromycin (10 μg/mL, MCE, USA). PPARγ expression level was routinely tested by Western blot and qRT-PCR analyses.

### Single-cell RNA sequencing preparation

The brain dissection and cell dissociation protocol for mice was performed as previously described 89 and conducted in six independent experiments (Aβo group and Mock group, n = 3/group). Mice were anesthetized with isoflurane, and their brains were rapidly removed and washed in ice-cold D-PBS (Hyclone, USA). Tissue dissociation was then performed using the Adult Brain Dissociation kit (Miltenyi Biotec, Germany) according to the manufacturer's instructions. Briefly, whole-brain tissue from each sample was loaded into a gentleMACS C tube with Enzyme mix 1 and Enzyme mix 2 and dissociated using the gentleMACS Octo Dissociator with Heaters for 20-100 mg of tissue. After dissociation, the samples were strained through a 70 μm filter and washed with D-PBS, followed by centrifugation at 300 g for 10 min at 4°C. The supernatant was removed, and the tissue was resuspended in an appropriate amount of D-PBS ( < 400 mg, 1550 μL). Debris Removal Solution was then added (< 400 mg, 450 μL), and the sample was covered with D-PBS, ensuring the lower layer remained undisturbed. The samples were centrifuged at 3000 g for 10 min at 4°C. After removing the upper two layers of liquid, fresh D-PBS was added, and the cells were centrifuged at 1000 g for 10 min. The cell pellet was carefully resuspended in an appropriate amount of cold 1× Red Blood Cell Removal Solution and incubated for 10 min at 4°C. After incubation, cold PB buffer was added, and the sample was centrifuged at 300 g for 10 min at 4°C. The supernatant was aspirated completely and the cells were resuspended in D-PBS containing 0.5% BSA. Cell count and viability were assessed using Countstar® Rigel S2 Fluorescence Cell Analyzer (Countstar, China) with AO/PI reagent. The results indicated that the cell viability in all samples was greater than 90%. Subsequently, the cell concentration of each sample was then adjusted to 1 × 10^6^ cells/mL for further single-cell capture. Throughout the process, both the tissue and cells were maintained in cold solution.

### Single cell RNA sequencing library construction and sequencing

Single-cell RNA-Seq libraries were prepared using SeekOne® Digital Droplet Single Cell 3' library preparation kit (SeekGene, China). Briefly, the appropriate number of cells obtained above were mixed with reverse transcription reagent and then added to the sample well in SeekOne® chip S3. Subsequently barcoded hydrogel beads (BHBs) and partitioning oil were dispensed into corresponding wells separately in chip S3. After emulsion droplet generation reverse transcription was performed at 42℃ for 90 min and inactivated at 85℃ for 5 min. Next, cDNA was purified from broken droplet and amplified in PCR reaction. The amplified cDNA product was then cleaned, fragmented, end repaired, A-tailed and ligated to sequencing adaptor. Finally, the indexed PCR was performed to amplify the DNA representing 3' polyA part of expressing genes which also contained Cell Barcode and Unique Molecular Index. The indexed sequencing libraries were cleaned up with VAHTS DNA Clean Beads N411-01 (Vazyme, China), analysed by Qubit Q33226 (Thermo Fisher Scientific, USA) and Bio-Fragment Analyzer Qsep400 (Bioptic, China). The libraries were then sequenced on Illumina NovaSeq X Plus (Illumina, USA) with PE150 read length.

### Single-cell RNA sequencing analysis

We used the Seekone Tools v1.2.1 (SeekGene, China) pipeline to process the cleaned reads and generate the transcript expression matrix. Briefly, raw sequencing data were demultiplexed, aligned to the mouse genome (mm10), and gene-specific read counts were generated. Downstream analysis was conducted using the Seurat R package (v4.4.0) within the R environment (v4.3.0). The data processing involved filtering out the top and bottom 5% of cells based on feature counts, as well as excluding cells with more than 10% mitochondrial RNA reads, in order to eliminate low-quality cells and potential doublets. After log normalization of expression, samples were integrated. Principal component analysis (PCA) captured over 99% of the variability in the first 30 principal components (PCs). Clustering was performed using these 30 PCs, and the results were visualized with Uniform Manifold Approximation and Projection (UMAP).

Cell clusters were manually annotated to specific cell types based on literature and the mouse cell atlas dataset. Differentially expressed genes (DEGs) between groups within each cluster were identified using the FindAllMarkers function. GO and KEGG pathway enrichment analysis of these DEGs was performed using clusterProfiler 4.0, and relevant terms were selected and visualized in the resulting plots.

To define phagocytosis score, we constructed a gene set associated with phagocytic function based on using annotations from GO:0006909 (phagocytosis) and KEGG:mmu04145 (Phagosome). Phagocytosis score was evaluated with the *AddModuleScore* function built in the Seurat 90. We first performed a Kruskal-Wallis test to assess phagocytosis score differences between microglial subgroups. A pairwise Wilcoxon rank-sum test was then conducted to compare GeneRatio cluster 3 with other groups. Multiple group comparisons were corrected using Dunn's test with Bonferroni adjustment to control for type I error.

Cell-cell communication analysis was performed using the CellChat R package (version 1.6.1). After preprocessing scRNA-seq data and assigning cell identities, intercellular signaling in Cluster 3 was inferred based on a ligand-receptor database. Significant signaling pathways were identified and visualized, and comparisons between the Sham and Aβo groups were made using the compareInteractions and rankNet functions, with default parameter settings throughout.

### RT-qPCR

RNA was extracted from primary microglial cell cultures or mouse brain samples using TRIzol reagent (Invitrogen, USA) following the manufacturer's protocol. RNA samples were converted to cDNA by HiScript^®^III RT SuperMix Kit (Vazyme, China) according to the manufacturer's protocol. RT-qPCR was performed in CFX96 real-time PCR machine (BIORAD, USA) using ChamQ Universal SYBR qPCR Master Mix (Vazyme, China). The 2^-ΔΔCt^method was used to calculate mRNA expression relative to *GAPDH*. A complete list of primer sequences is provided in Table S3.

### Western blot

Brain tissues or cultured cells were homogenized in RIPA lysis buffer (Biosharp, China) containing protease inhibitor, and protein concentration was quantified by BCA protein assay kit (Beyotime, China) following manufacturer's instructions. The protein samples were then loaded and separated by SDS-PAGE on 10% tris-glycine gels before being transferred onto polyvinylidene difluoride (PVDF) membranes (Roche Diagnostics, USA). Then 5% nonfat milk was used to block the membranes for 2h at room temperature before incubation with different primary antibodies overnight at 4°C. Primary antibodies were used as follows: Rabbit anti-Mertk (1:1000, ab95925, abcam, UK); Rabbit anti-PPARγ (1:1000, 16643-1-AP, Proteintech, China); Mouse anti-β-actin (1:50000, 66009-1-Ig, Proteintech, China); Mouse anti-GAPDH (1:50000, 60004-1-Ig, Proteintech, China). After washing, the membranes were incubated with horseradish peroxidase (HRP)-conjugated secondary antibodies for 2h at room temperature. Secondary antibodies were used as follows: HRP-conjugated Goat Anti-Rabbit (1:10000, SA00001-2, Proteintech, China); HRP-conjugated Goat Anti-Mouse (1:10000, SA00001-1, Proteintech, China). Then ECL Western Blotting Detection Kit (Thermo Fisher Scientific, USA) was used for color detection, and ImageJ software was used to carry out densitometry analysis of bands.

### Immunohistochemistry

Mice were anaesthetized using isoflurane and perfused with an intracardiac injection of phosphate-buffered saline (PBS) sequentially. Brains were post-fixed in 4% paraformaldehyde (PFA) at 4°C overnight, then washed and soaked in 30% sucrose solution for dehydration. After embedding brain tissues with optimal cutting temperature compound, 20 μm thick coronal slices from the hippocampus were prepared by Leica cryostat (Leica CM1950, Germany). Afterwards, tissue sections were blocked with 5% bovine serum albumin (BSA, Beyotime, China) with 0.3% triton-X100 (Beyotime, China) in PBS for 1 h, and incubated with primary antibodies overnight. Primary antibodies were used as follows: Rabbit anti-IBA1 (1:1000, ab178846, abcam, UK); Mouse anti-IBA1 (1:500, ab283319, abcam, UK), Goat anti-Mertk (1:200, AF591, R&D systems, USA); Rabbit anti-PSD95 (1:200, 51-6900, Invitrogen, USA); Mouse anti-Synapsin I (1:400, 106011, Synaptic Systems, Germany). After washing with PBS, appropriate secondary fluorescent antibodies were incubated for 2h at room temperature. Secondary antibodies were used as follows: Goat anti-Mouse Alexa Fluor™ 647 (1:1000, A21236, Invitrogen); Goat anti-Rabbit Alexa Fluor™ 488 (1:1000, A11008, Invitrogen); Donkey anti-Rabbit Alexa Fluor™ Plus 488 (1:1000, A32790, Invitrogen); Donkey anti-Goat Alexa Fluor™ Plus 594 (1:1000, A32758, Invitrogen). Images were acquired using Leica TCS SP8 or Zeiss LSM980 confocal microscope. For primary cultured cell immunostaining, cells were fixed with 4% PFA for 30 min, and the following procedure was the same as brain tissues.

### Synapse quantifications

Quantification of synaptic puncta was based on a published study with several modifications 44. Briefly, 20 μm sections from the hippocampus stained for pre-synaptic proteins (Synapsin I) and post-synaptic proteins (PSD95). Super-resolution 4 μm z-stack comprised 0.2 μm z-steps images were acquired using a Zeiss LSM980 confocal microscope with Airyscan 2 (Carl Zeiss, Germany) at 63x oil immersion objective. Each field (8-10 images total for each animal) was imaged. Afterwards, pre-synaptic and post-synaptic puncta were sequentially rendered in 3D using the surface function on Imaris 9.6.1 software (Bitplane, Switzerland). The number of colocalized pre-synaptic and post-synaptic puncta was analysed using the "Surface-surface colocalization" of Imaris plugin. This XTension finds the overlapping voxels within each surface and generate a new surface from these regions, which can then be rendered and analysed. Synaptic density was determined as puncta number/given area. The representative figures were compiled by Imaris 9.6.1 software. Imaging and analysis were performed blind to genotype.

### *In vivo* engulfment assay with microglia

For microglial engulfment assay, 20 μm brain sections from the hippocampus stained with Iba-1 were imaged using Leica TCS SP8 confocal microscope at 63x oil immersion objective with 0.25 μm z-step. Images were processed and analysed as described previously 32. Briefly, 3D surface renderings of the microglia and synaptic puncta of each z-stack were created using Imaris 9.6.1 software (Bitplane, Switzerland). The "surface-surface colocalization" of the Imaris plugin was used to analyze synaptic puncta internalized by microglia (Video S9). Afterwards, Microglial engulfment percentage was calculated as volume of internalized synaptic puncta/volume of microglial cell. The representative figures were compiled by Imaris 9.6.1 software. Imaging and analysis were performed blind to genotype.

### Synaptosome isolation and pHrodo labeling

Synaptosomes were purified by percoll gradient from the adult mouse brains as described previously 91. Briefly, fresh brain tissue was homogenized in ice-cold homogenization buffer (0.32 M sucrose, 1 mM EDTA, 0.25 mM DTT and 5 mM Tris, pH = 7.4) and then the brain extracts were centrifuged at 1000 g for 10 min at 4 °C. The supernatants were added to the 3% layer in a tube containing the Percoll gradient solutions (3%, 10%, 15%, and 23%) and the tube was centrifuged at 31,000 g for 5 min at 4°C using the Beckman Coulter Allegra 64R (Beckman Coulter, USA). Two fractions were collected from the interface between the 10% and 23% Percoll layer. The collected samples were diluted with a four-fold volume of ice-cold sucrose/EDTA buffer to remove the Percoll and then centrifuged at 20,000 g for 30 min at 4 °C. The obtained synaptosome was incubated with pHrodo™ iFL red STP ester (P36011, Thermo Fisher Scientific, USA) in 0.1 M sodium carbonate (pH 9.0) at room temperature in the dark at a concentration of 1 μL pHrodo/1 mg synaptosomes with gentle agitation 92. After 2h incubation, unconjugated pHrodo was washed out by multiple rounds of centrifugation and pHrodo-conjugated synaptosomes was then added to primary microglia cultures to evaluate the phagocytosis of microglia.

### *In vitro* engulfment assay with microglia

Phagocytosis assay was performed based on the protocol previously described 93. Latex beads (1 μM, L2778, Sigma-Aldrich, USA) were opsonized with complete medium (10% FBS in DMEM) for 1 h at 37 °C before the experiments. Opsonized beads were added to Aβo-treated primary microglia at a 10:1 ratio and incubated at 37 °C for 2 h. Next, primary microglia were washed three times with PBS to remove non-phagocytized beads and fixed with 4% paraformaldehyde at room temperature. Immunofluorescence was then performed, and primary microglia were visualized using a Leica LCS SP8 STED confocal laser-scanning microscope (Leica Microsystems, Germany) with a 63x oil immersion objective (Video S10). Quantitative analysises of microglial phagocytosis capacity were expressed as the ratio of total area of fluorescent signals to the total number of phagocytic cells. Imaging and analysis were performed blind to genotype.

To realistically simulate microglia phagocytic synapses *in vitro*, primary microglial cells were pretreated with Aβo for 24 h, followed by incubation with pHrodo-conjugated purified synaptosomes. The phagocytosis of pHrodo-conjugated particles was imaged for 6 h using High-Content Live-Cell Imaging (Video S1-8). Images should be captured while the cells were alive, as the fluorescence of pHrodo-red is undetectable after fixation. For image analysis, 6-8 images per well were captured using a 40x objective lens from random areas of the 24-well plates. The ability of microglia to phagocytize synapses was evaluated by the mean fluorescence intensity and the phagocytic index (PI). PI was determined by calculating the fraction of the microglia area that was overlapped by the pHrodo area (engulfed synaptosomes) using FIJI. Imaging and analysis were performed blind to genotype.

### Electrophysiology

After anesthesia with sodium pentobarbital, the heads of mice were harvested, and the brain tissue was carefully trimmed and placed in a slicing chamber. Coronal slices containing the hippocampus were obtained at a thickness of 300 µm using a vibroslicer set to a speed of 0.22 mm/s and an amplitude of 0.85 mm. The slices were incubated in artificial cerebrospinal fluid (ACSF) composed of NaCl 124 mM, KCl 2.5 mM, D-glucose 10 mM, CaCl_2_ 0.5 mM, MgCl_2_·6H_2_O 1.3 mM, NaHCO_3_ 26 mM, NaH_2_PO_4_ 1.25 mM, adjusted to pH 7.3 with HCl, which was equilibrated with 95% O_2_ and 5% CO_2_, at 32 ± 0.5 °C for at least 30 min. Subsequently, the slices were stored at room temperature. mEPSCs were recorded using electrodes with resistances ranging from 4-7 MΩ. The internal solution for the electrodes comprised 122 mM K-Gluconate, 0.4 mM Na_2_-GTP, 5 mM Na_2_-ATP, 5 mM NaCl, 1 mM EGTA, 10 mM HEPES, 0.3 mM CaCl_2_, 2 mM MgCl_2_·6H_2_O, adjusted to pH 7.3 by KOH. The external solution contained 10 μM Bicuculline and 1 μM tetrodotoxin (MedChen Express). All currents were recorded at a holding potential of -70 mV using an Axopatch-700B amplifier (Axon Instruments, CA). Data analysis was performed using Clampfit 10.2 software.

### Morris water maze test

The Morris water maze test was performed as previously described 71. Briefly, each mouse was trained consecutively for 5 days, receiving training in each of the four quadrants of the pool to locate a hidden platform underwater. Time taken by the mouse to find and climb onto the platform was recorded as latency period. On the sixth day of spatial probe trial, the platform was removed from the pool, and the mice were allowed to swim for 60 s. The trajectory of mice in the pool, the latency required to reach original platform location, and the number of crossings over the platform location were recorded by water maze software (SMART, USA) for analysis.

### Y maze test

The mice were placed in a Y-shaped maze consisting of three arms of equal length, which diverged at equal angles as previously detailed 59. Mice were individually placed at the junction of the arms and allowed to move freely for 8 min. The total number and order of entries to the arm during the mice test were recorded. A successful spontaneous alternation was defined as entering all three arms consecutively without returning to any previously visited arm. The percentage of spontaneous alternations was calculated as (the number of spontaneous alternations/the total number of arm entries minus two) × 100.

### ChIP assay

Briefly, BV2 stable cell lines overexpressing PPARγ or transfected with control vector were seeded into 100 mm cell culture dishes. Then, cells were collected and processed according to the instructions of the ChIP assay kit (Beyotime, China). The resulting DNA products were purified using Universal DNA Purification Kit (TIANGEN, China). The primers for quantitative PCR were designed to target specific regions predicted to bind by the website. Quantitative PCR was performed on DNA samples extracted from ChIP to assess the binding of PPARγ to the Mertk promoter region. The ChIP-PCR primers used in this experiment are listed in Table S4.

### Dual-luciferase reporter assay

HEK293T cells were seeded in 24-well plates and transfected with reporter gene plasmids pGL3-Mertk (300 ng/well, GENE CREATE, China), the overexpression plasmid pCMV-PPARγ (1 μg/well, miaolingbio, China) and pRL-TK plasmids (2 ng/well, GENE CREATE, China) using Lipofectamine 2000 reagent (Invitrogen, USA), following the manufacturer's protocol. After 48 h of transfection, cells were harvested and the reporter gene activity of each group was measured on GloMax20/20 Luminometer system (Promega, USA) using Dual-Luciferase Reporter Gene Assay Kit (Beyotime, China) according to the instructions.

### Statistical analysis

All individual measurements constitute biological replicates. The charts and statistical analyses were generated using GraphPad Prism 8.3.0 software, with all values represented as mean ± standard error of the mean (SEM). All data were confirmed to follow a normal distribution by Shapiro-Wilky tests. Data with two groups were analysed using Student's *t*-test for normally distributed continuous variables; otherwise, the Mann-Whitney test was employed. For comparisons of multiple groups, data were analysed by one-way ANOVA (one-factor analysis) followed by Newman-Keuls post-tests or two-way ANOVA (two-factor analysis) followed by Bonferroni post-tests. Statistical significance was denoted by *P < 0.05, **P < 0.01, or ***P < 0.001.

## Supplementary Material

Supplementary figures and tables.

Supplementary video 1-4.

Supplementary video 5-8.

Supplementary video 9.

Supplementary video 10.

## Abbreviations

AD: Alzheimer's disease
Aβ: amyloid-beta
Aβo: amyloid-beta oligomers
ChIP: chromatin immunoprecipitation
CNS: central nervous system
Dual-LUC: dual-luciferase reporter assay
Gas6: growth arrest-specific gene 6
GEO: Gene Expression Omnibus
GW: PPARγ-selective inhibitor GW9662
ICV: intracerebroventricular
IF: immunofluorescence
IP: intraperitoneal injection
KEGG: Kyoto Encyclopedia of Genes and Genomes
MCI: mild cognitive impairment
mEPSCs: miniature excitatory postsynaptic currents
Mertk: Mer tyrosine kinase
OE: overexpression
PI: phagocytic index
PPARγ: peroxisome proliferator-activated receptor gamma
Pros1: protein S
PSD95: postsynaptic density protein 95
PtdSer: phosphatidylserine
scRNA-seq: single-cell RNA sequencing
SEM: standard error of the mean
TAM: Tyro3, Axl, and Mertk receptor family
TSS: transcription start site
UMAP: uniform manifold approximation and projection
UNC: Mertk-selective antagonist UNC2250
WT: wild type
