# Supplementary material for: Mertk promotes early microglial-mediated synaptic engulfment in Alzheimer's disease
Source: Theranostics. 2026 Jan 1;16(3):1238–61. doi: 10.7150/thno.116797 (PMC12679373; doi:10.7150/thno.116797)
Supplement: Supplementary file 1 — Supplementary figures and tables. [file thnov16p1238s1.pdf]

Mertk promotes early microglial-mediated synaptic engulfment in Alzheimer's disease

Supplementary figures and tables

Figure S1. GO and KEGG pathway analysis of differentially expressed genes in cluster 3 microglia

Figure S2. Increased microglia before Aβ plaque formation in the hippocampus of 4-month-old 3xTg-AD mice.

Figure S3. Early Mertk expression and microglial synaptic engulfment in AD mice.

Figure S4. Correlation between Mertk expression and clinical cognitive function in AD patients.

Figure S5. Genetic deletion or pharmacological inhibition of Mertk alleviates cognitive impairment in AD mice.

Figure S6. Downregulation of phosphorylated tau protein in the brains of aged Mertk$^{-/-}$ mice.

Figure S7. Quantification of synaptosomes engulfed by primary microglia.

Figure S8. Inhibiting PPARγ alleviates cognitive impairment in 3xTg-AD mice.

Figure S9. The knockout status of Mertk.

Video S1-4. High-content live-cell imaging reveals phagocytosed synaptosomes (orange) engulfed by primary microglia in different groups (WT, Mertk$^{-/-}$, WT+Aβo, Mertk$^{-/-}$+Aβo) in real time over a 6-hour period.

Video S5-8. High-content live-cell imaging reveals phagocytosed synaptosomes (orange) engulfed by primary microglia in different groups (Mock, GW, Aβo, Aβo+GW) in real time over a 6-hour period.

Video S9. 3D rendering of synapses engulfed by microglia using the Imaris "Surface-Surface Colocalization" plugin.

Video S10. 3D rendering of latex beads engulfed by microglia using the Imaris "Surface-Surface Colocalization" plugin.

Table S1. PCR primers for Mertk knockout identification.

Table S2. Information of human samples.

Table S3. PCR primers sequence.

Table S4. ChIP-PCR primers sequence.

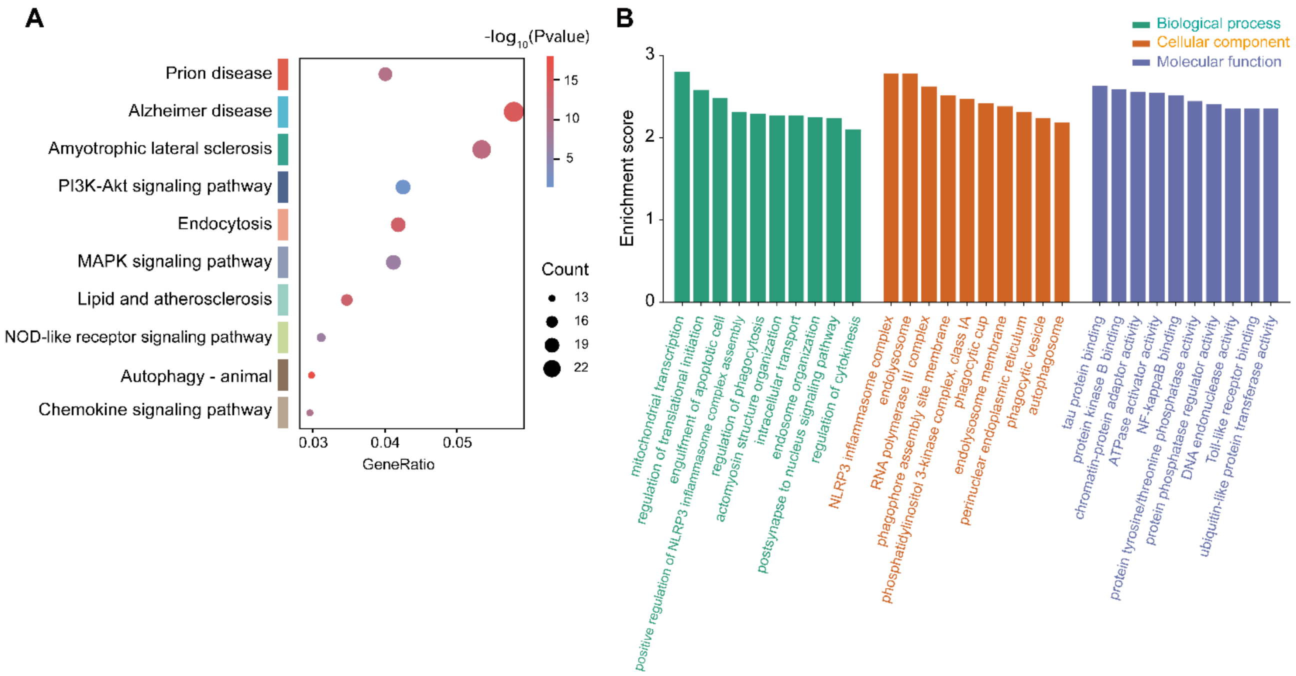

Figure S1. KEGG and GO enrichment analyses of differentially expressed genes in cluster 3 microglia. (**A**) KEGG pathways. (**B**) GO terms.

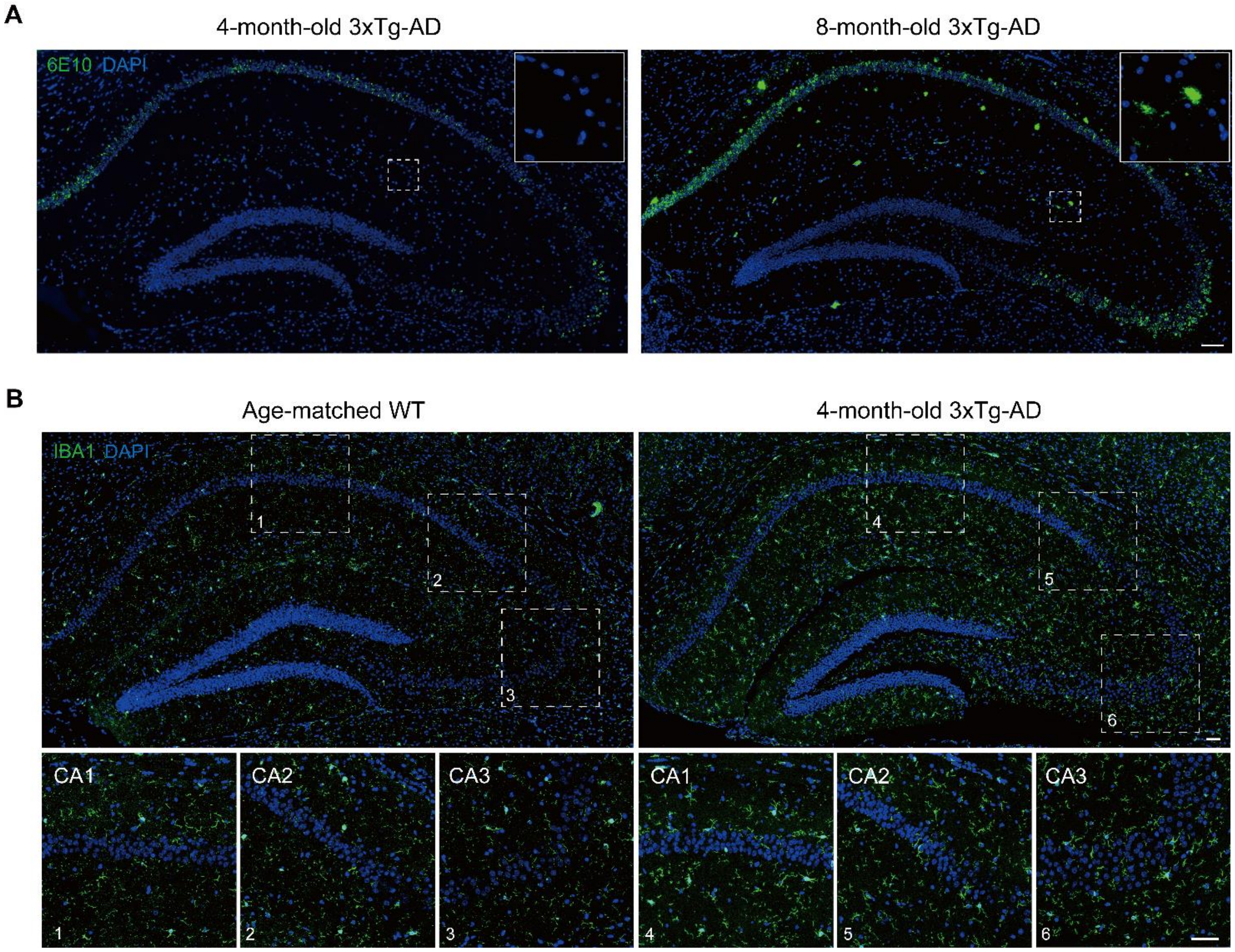

Figure S2. Increased microglia before Aβ plaque formation in the hippocampus of 4-month-old 3xTg-AD mice. (**A**) Representative images of 6E10 immunoreactive plaques in 4-month-old and 8-month-old 3xTg-AD mice. Scale bar, 100 μm. (**B**) Immunofluorescence staining showed an increased number of microglia ($IBA1^{+}$) in the hippocampus of 4-month-old 3xTg-AD compared to age-matched WT mice. Scale bar, 50 μm.

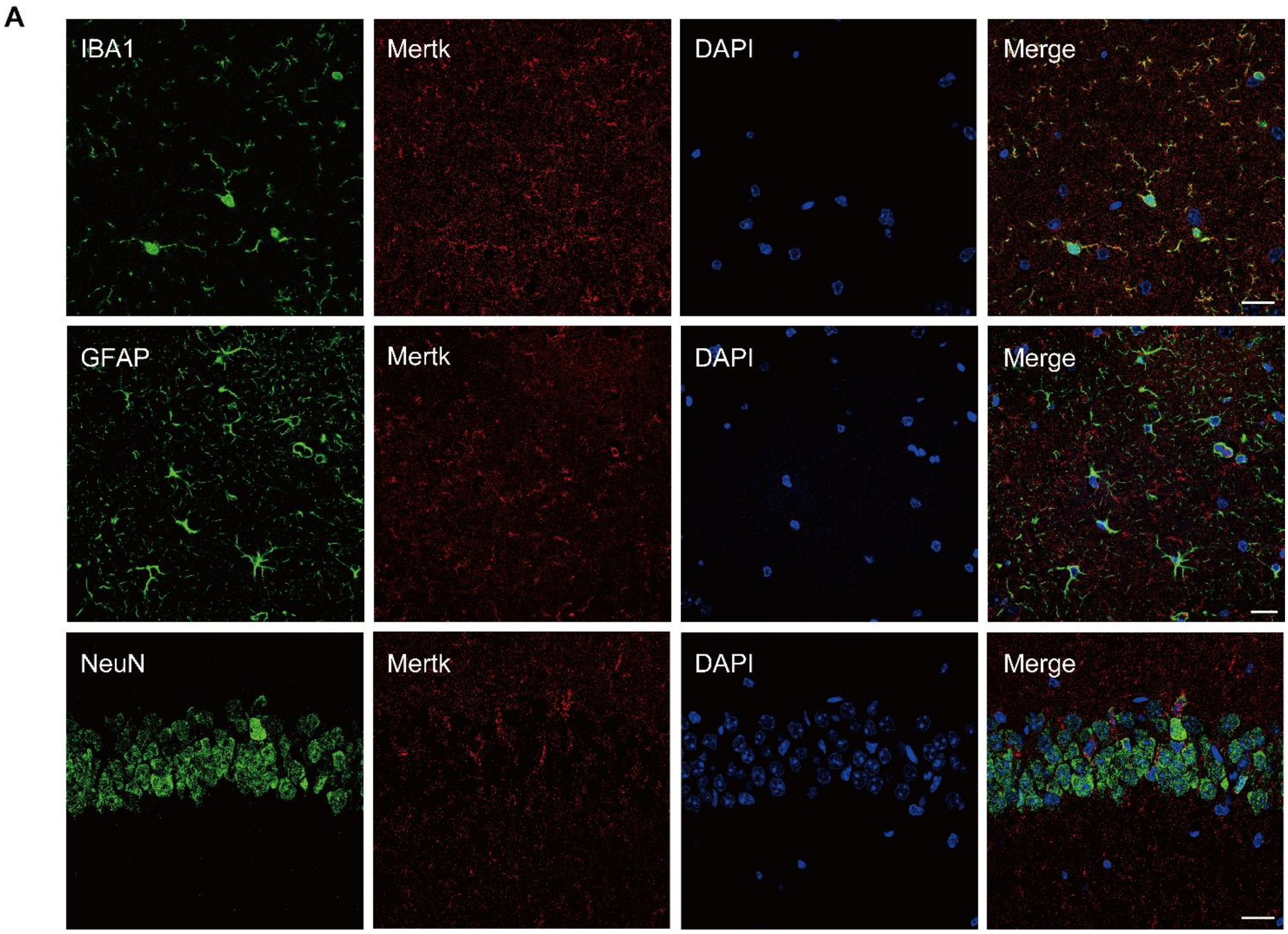

Figure S3. Early Mertk expression and microglial synaptic engulfment in AD mice. (**A**) Mertk is primarily expressed in microglia (IBA1^{+}), but not in astrocyte (GFAP^{+}) and neuron (NeuN^{+}) in the hippocampus of AD mice. Scale bar: 20 μm. These experiments were independently repeated three times.

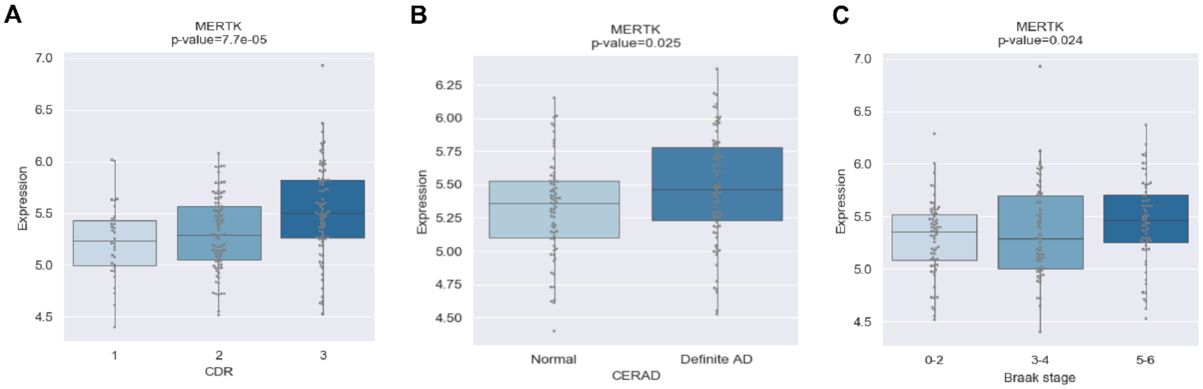

Figure S4. Correlation between Mertk expression and clinical cognitive function in AD patients. (**A**) Correlation between Mertk expression and Clinical Dementia Rating (CDR) scale. (**B**) Correlation between Mertk expression and Consortium to Establish a Registry for Alzheimer's Disease (CERAD) scores. (**C**) Correlation between Mertk expression and Braak staging.

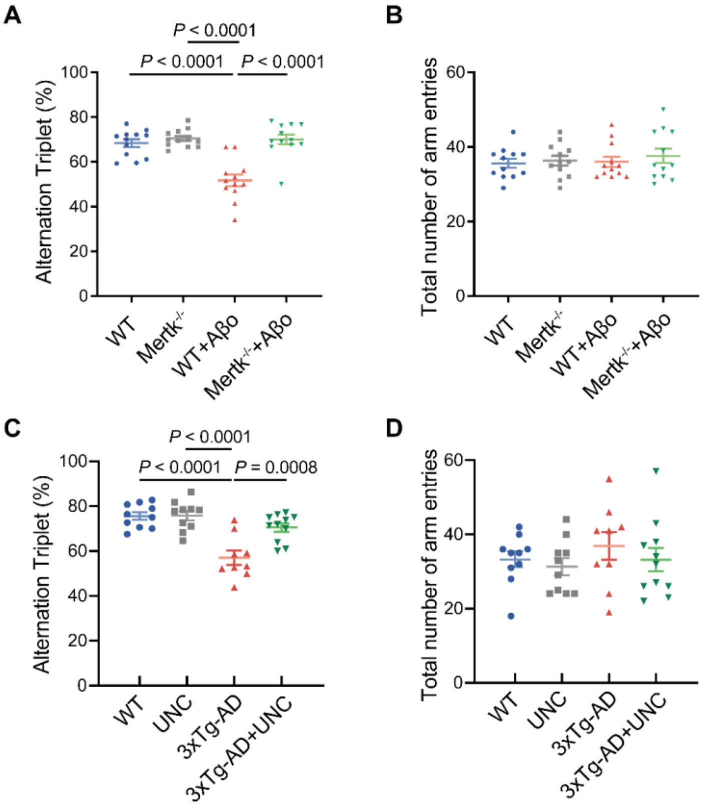

Figure S5. Genetic deletion or pharmacological inhibition of Mertk alleviates cognitive impairment in AD mice. (**A, B**) Y maze assay results: (A) percentage of spontaneous alternations and (B) total arm entries in the four groups (WT, Mertk$^{-/-}$, WT+AβO and Mertk$^{-/-}$+AβO; *n* = 12 mice/group; one-way ANOVA with Dunnett's multiple comparisons test). (**C, D**) Y maze assay results: (C)percentage of spontaneous alternations and (D) total arm entries across the four groups (WT, UNC, 3xTg-AD, 3xTg-AD+UNC; *n* = 10, 10, 9, 11 mice/group; one-way ANOVA with Dunnett's multiple comparisons test). Data are presented as means ± SEM.

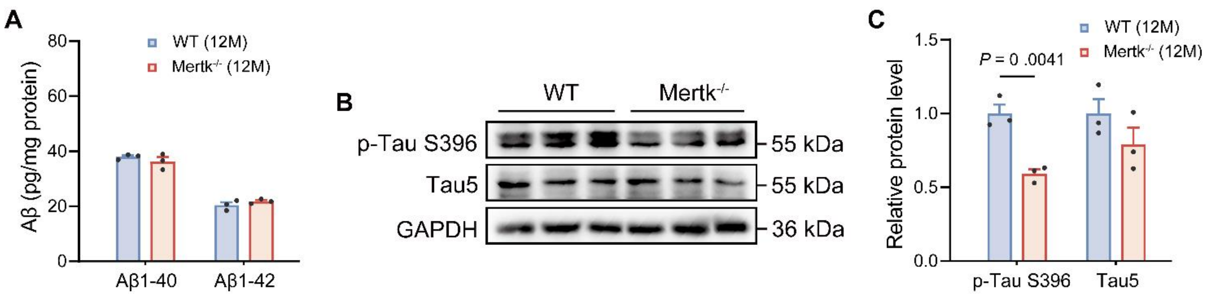

Figure S6. Downregulation of phosphorylated tau protein in the brains of aged Mertk$^{-/-}$ mice. (**A**) ELISA quantification of Aβ1-40 and Aβ1-42 levels in the brains of 12-month-old Mertk$^{-/-}$ and WT mice. *n* = 3 mice/group; two-tailed unpaired *t*-test. (**B, C**) Western blot analysis of phosphorylated tau (pS396) and total tau (Tau5) protein levels in 12-month-old Mertk$^{-/-}$ and WT mice. *n* = 3 mice/group; two-tailed unpaired *t*-test.

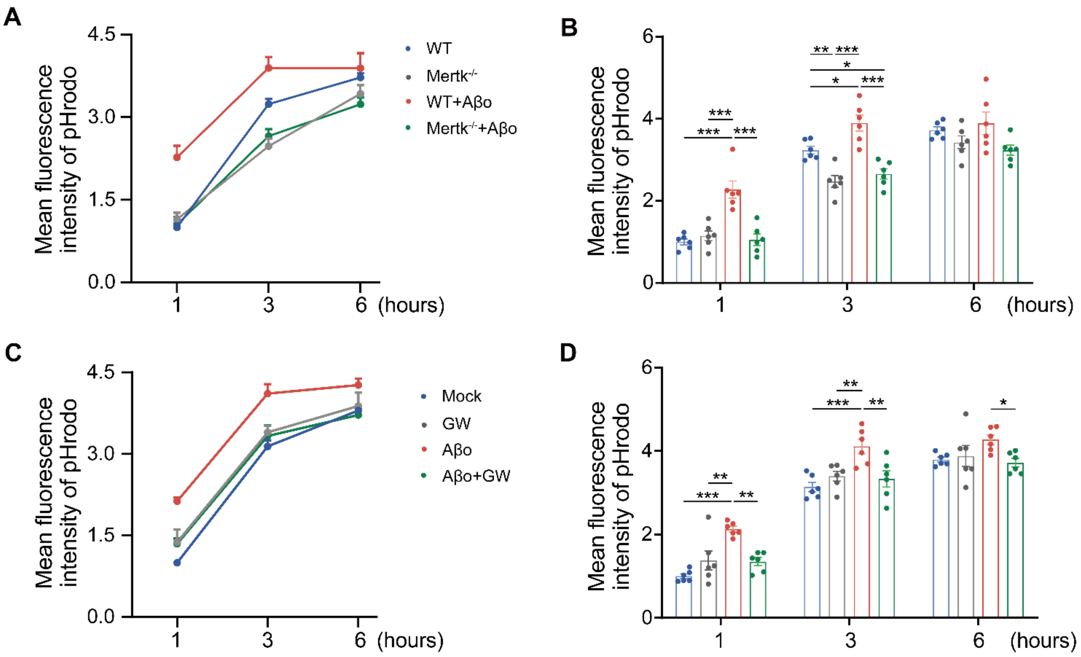

Figure S7. Quantification of synaptosomes engulfed by primary microglia. (**A, C**) Normalized mean fluorescence intensity was used to quantify the number of phagocytic synaptosomes over time. (**B, D**) The histogram shows the average fluorescence intensity in different groups at representative time points. (A, B) WT, Mertk$^{-/-}$, WT+AβO, Mertk$^{-/-}$+AβO; (C, D) Mock, GW, Aβo, Aβo+GW. $n = 6$ wells; average of 6–8 fields from each well were analyzed using two-way ANOVA with Tukey's multiple comparisons test. Data are presented as means ± SEM. $^{*}P < 0.05$, $^{**}P < 0.01$, $^{***}P < 0.001$.

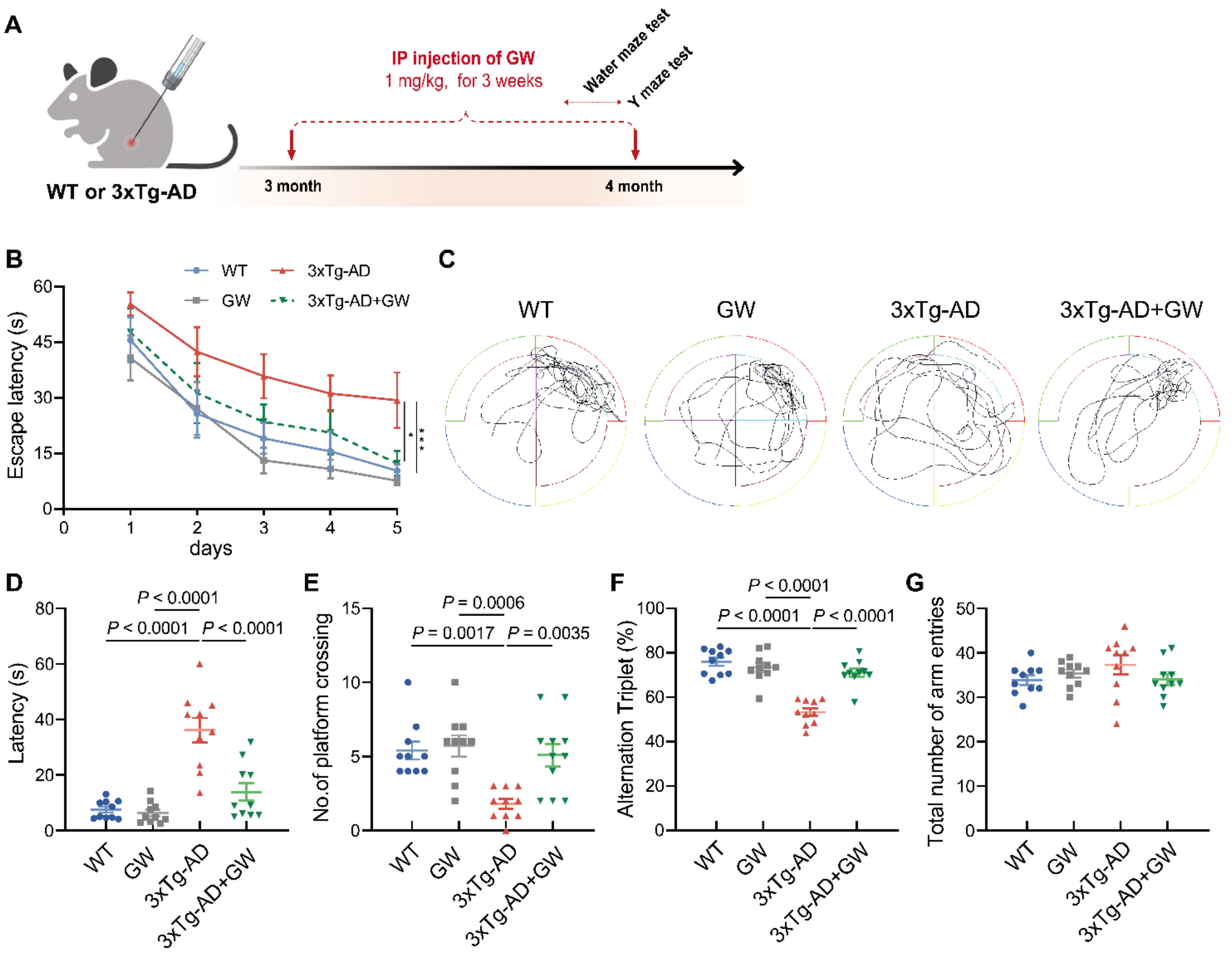

Figure S8. Inhibiting PPARγ alleviates cognitive impairment in 3xTg-AD mice. (**A**) Experimental schematic showing treatment and testing timeline. IP: intraperitoneal injection; GW: GW9662. (**B**) Line chart of Morris water maze escape latency over five training days in the four groups (WT, GW, 3xTg-AD, 3xTg-AD+GW; *n* = 10 mice/group; two-way repeated measures ANOVA with Tukey's multiple comparisons test). (**C**) Representative trajectory of the four groups during Morris water maze spatial probe trial. (**D, E**) Graph of the time spent to reach original platform location (D) and the number of platform crossings (E) during the spatial probe trial. (*n* = 10 mice/group; one-way ANOVA with Dunnett's multiple comparisons test). (**F, G**) Y maze assay results: (F) percentage of spontaneous alternations and (G) total arm entries across the four groups (*n* = 10 mice/group; one-way ANOVA with Dunnett's multiple comparisons test). Data are presented as means ± SEM. **P* < 0.05, ****P* < 0.001.

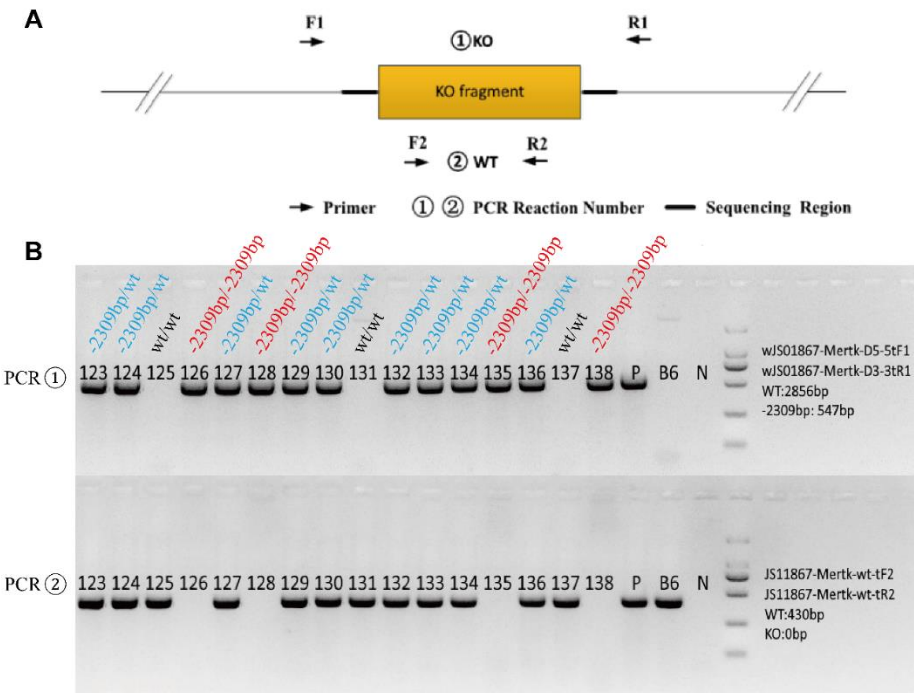

Figure S9. The knockout status of Mertk. (**A**) Identification scheme. (**B**) The sizes of WT and Targeted band are shown. Wild type: PCR① reaction obtains a single WT band; PCR② reaction obtains a single WT band. Heterozygote: PCR① reaction obtains a WT band and a KO band; PCR② reaction obtains a WT band. Homozygote: PCR① reaction obtains a single KO band; PCR② reaction without product. If the WT band is too large, it may not be possible to obtain a WT band.

Video S1–4. High-content live-cell imaging reveals phagocytosed synaptosomes (orange) engulfed by primary microglia in different groups (WT, Mertk$^{-/-}$, WT+Aβo, Mertk$^{-/-}$+Aβo) in real time over a 6-hour period.

Video S5–8. High-content live-cell imaging reveals phagocytosed synaptosomes (orange) engulfed by primary microglia in different groups (Mock, GW, Aβo, Aβo+GW) in real time over a 6-hour period.

Video S9. 3D rendering of synapses engulfed by microglia using the Imaris "Surface-Surface Colocalization" plugin.

Video S10. 3D rendering of latex beads engulfed by microglia using the Imaris "Surface-Surface Colocalization" plugin.

Table S1. PCR primers for Mertk knockout identification.

PCR No.	Primer Name	Sequence	Band Size
PCR①	wJS01867-Mertk-D5-5tF1	TAGCAACCAGCAGAGAATGTGTG	WT: 2856bp
	wJS01867-Mertk-D3-3tR1	GGTGAGTCAGACTAAGGAGCTACC	-2309bp: 547bp
PCR②	JS11867-Mertk-wt-tF2	AGGCAGCAAGTACACAGAGTGAGG	WT: 430bp
	JS11867-Mertk-wt-tR2	GCTGATCTAGCTCGGTCTCTTCC	KO: 0bp

Table S2. Information of human samples.

Case No	Diagnosis	Sex	Age (years)	Braak stage	ApoE
1	control	male	70	I	E3/3
2	control	male	69	II	E3/3
3	control	female	60	I	E3/3
4	control	female	79	I	E3/3
5	AD	male	69	VI	E3/3
6	AD	female	57	V	E3/4
7	AD	male	79	IV	E3/3
8	AD	female	79	IV	E3/3

Table S3. PCR primers sequence.

Gene	Forward primer (5′-3′)	Reverse primer (5′-3′)
Mertk	CTCCTGAGCCCGTCAATATCT	AGACCAGGTACGGTTAGGACA
Sp1	AGGGTCCGAGTCAGTCAGG	CTCGCTGCCATTGGTACTGTT
Sp3	TCAAGTAGTCGCTAATGTGCCT	GAACTTCCCGAGAGTCCCAAA
E2f1	TGCAGAAACGGCGCATCTAT	CCGCTTACCAATCCCCACC
PPARγ	TTTTCCGAAGAACCATCCGATT	ATGGCATTGTGAGACATCCCC
GAPDH	TCGCTCCTGGAAGATGGTGAT	CAGTGGCAAAGTGGAGATTGTTG

Table S4. ChIP-PCR primers sequence.

Region	Forward primer (5′-3′)	Reverse primer (5′-3′)
-50 to -250 bp	CACTCAAGCCAGGGGTTAAC	CCAGCAGGTTGGAGTGGA
-200 to -400 bp	AGTAGCCAGAGATCATCCTCC	GCAGAATGGAACTTGGGTCAA
-300 to -500 bp	CTCCACTAAATTCGGCCTCTAC	GGAGGATGATCTCTGGCTACT
-650 to -850 bp	CTCATACGCCTTCCGTTTCT	GCCGAATCCTGTAAGGAAAGA
